# Efficacy and safety of monotherapy and combination therapy of immune checkpoint inhibitors as first-line treatment for unresectable hepatocellular carcinoma: a systematic review, meta-analysis and network meta-analysis

**DOI:** 10.1007/s12672-022-00559-1

**Published:** 2022-09-28

**Authors:** Qing Lei, Xin Yan, Huimin Zou, Yixuan Jiang, Yunfeng Lai, Carolina Oi Lam Ung, Hao Hu

**Affiliations:** 1 State Key Laboratory of Quality Research in Chinese Medicine, Institute of Chinese Medical Sciences, University of Macau, Macao SAR, China; 2 School of Public Health and Management, Guangzhou University of Chinese Medicine, Guangzhou, China; 3 Department of Public Health and Medicinal Administration, Faculty of Health Sciences, University of Macau, Macao SAR, China

**Keywords:** Unresectable hepatocellular carcinoma, First-line, Immune checkpoint inhibitors, Meta-analysis, Network meta-analysis

## Abstract

**Background:**

Hepatocellular carcinoma (HCC) is one of the cancers with the highest morbidity and mortality. Sorafenib used to be the main treatment for unresectable HCC patients. However, regimens based on immune checkpoint inhibitors (ICIs) have attracted attention in recent years because of their reported benefits. This study aimed to evaluate the efficacy and safety of monotherapy and combination therapy of ICIs as first-line treatment for unresectable HCC patients by conducting a systematic review, meta-analysis, and network meta-analysis.

**Methods:**

Studies published up to 11st August 2022 were searched from 4 commonly used databases, including PubMed, Web of Science, Embase, and Clinical trials.gov. All eligible clinical trials were included. Data about reported objective response rate (ORR), disease control rate (DCR), overall survival (OS), progression-free survival (PFS), and treatment-related adverse events (TRAEs) were extracted.

**Results:**

Of the 8579 studies retrieved, 24 met the inclusion criteria. In patients with unresectable HCC taking ICIs-based therapy as first-line treatment, the pooled result of median PFS and median OS was 5.76 months (95% CI 4.82–6.69) and 16.35 months (95% CI 15.19–17.51) The ORR and DCR were 25.1% (95% CI 20.8–29.5%) and 75.2% (95% CI 70.3–80.2%) measured by RECIST v1.1 or 40.2% (95% CI 31.7–48.6%) with 75.2% (95% CI 68.3–82.1%) measured by mRECIST v1.1. Compared to sorafenib, ICIs-based therapy significantly prolonged OS. The combination treatment of sintilimab plus IBI305 had the highest ORR, while atezolizumab plus bevacizumab had the highest DCR. The pooled incidence of any grade TRAEs was 82.3% (95% CI 73.9–90.7%), with highest incidence appeared in dysphonia.

**Conclusions:**

This study demonstrated that first-line ICIs-based therapies could provide survival benefits for patients with unresectable HCC, with manageable TRAEs. The potential of combination treatment to become the new treatment trend in clinical practice is promising.

**Supplementary Information:**

The online version contains supplementary material available at 10.1007/s12672-022-00559-1.

## Introduction

Global cancer statistics 2020 reported that primary liver cancer was the sixth most common cancer and the third leading cause of cancer-related death worldwide, with hepatocellular carcinoma (HCC) accounting for 75–85% of primary liver cancer cases [1]. The age-standardized incidence rate of HCC was 7.3 cases per 100,000 person-year, including 11.6 cases per 100,000 in males and 3.4 cases per 100,000 in females [2]. The incidence rate of HCC had been increasing rapidly in recent decades, with a concomitant increase in mortality rate due to poor prognosis [3]. The main risk factors for HCC included cirrhosis, hepatitis B virus (HBV) and hepatitis C virus (HCV) infection, smoking, drinking, and consumption of aflatoxin-infected food. At the same time, overweight, diabetes and family history also increased the risk of HCC [4, 5].

Patients with early-stage or intermediate-stage HCC could be treated with local therapies, including liver resection, liver transplantation or trans-arterial chemoembolization (TACE). However, most patients were diagnosed only when they had reached the advanced stage. Before the clinical use of immunotherapies, tyrosine kinase inhibitors (TKIs) such as sorafenib and lenvatinib have been recommended to patients who received unresectable HCC in the last few years. Unfortunately, the prognosis for unresectable HCC patients remained unsatisfactory [6, 7]. Recently, the success of the use of immune checkpoint inhibitors (ICIs) in metastatic melanoma, lung cancer, and triple-negative breast cancer has brought new hope for unresectable HCC patients [8]. ICIs was one of the fastest-growing immunotherapeutic strategies and became a hot spot for anti-tumour drug research. Studies have reported good therapeutic effects of ICIs for solid tumours. Most of them blocked the relevant receptors to help T cells overcome the immune regulation and achieve anti-tumour effects [9].

As for unresectable HCC patients, several clinical trials showed that ICIs were effective and well-tolerated, and suggested that ICIs could be used as a treatment choice or in combination with other drugs [10–12]. In particular, the large clinical trial IMBrave150 reported that ICIs-based therapy, compared to sorafenib, resulted in significant benefits in terms of overall survival (OS) and progression-free survival (PFS) [13].

The National Comprehensive Cancer Network (NCCN) guidelines had included atezolizumab plus bevacizumab in the first-line treatment option for unresectable HCC and recommended the clinical use of immunotherapy for eligible patients [14].In addition, the Chinese Society of Clinical Oncology (CSCO) guidelines also recommended atezolizumab plus bevacizumab as well as other immunotherapies, such as lenvatinib plus camrelizumab or nivolumab, camrelizumab plus oxaliplatin-based chemotherapy, and camrelizumab plus apatinib as first-line treatment strategies for unresectable HCC patients [15].

At present, a research gap in comparing different ICIs-based regimens for unresectable HCC patients remains. Most of the available studies on the use of ICIs-based therapies for HCC patients only included some monotherapy or focused on both first-line and non first-line treatments at the same time [16–20]. With the rapid development of ICIs, evaluating them as first-line treatment in terms of efficacy and safety is needed to support evidence-based clinical practice.

Thus, we used systematic review, meta-analysis and network meta-analysis to evaluate the results of all clinical trials using ICIs-based treatment as first-line therapy for unresectable HCC patients. We hope that the results of this research could provide evidence for informing physicians’ choice of treatment and keeping researchers abreast of the latest evidence development in this field.

## Methods

This systematic review, meta-analysis and network meta-analysis complied with the PRISMA statement [21, 22]. The study protocol was also registered on the PROSPERO website, and the registration number was CRD42021288188.

### Inclusion and exclusion criteria

Inclusion criteria for clinical trials were developed in accordance with PICOS principles [23]. Clinical studies which investigated ICIs-based regimens for unresectable HCC were eligible for inclusion. The involved population must be adults with unresectable HCC who never received systematic treatment before. Patients with metastatic tumors were excluded from this study. Efficacy results included PFS, OS, objective response rate (ORR) and disease control rate (DCR), and the incidence of treatment-rated adverse events (TRAEs). The specific eligibility criteria for inclusion were shown in Table 1.

**Table 1:** Study inclusion and exclusion criteria

Characteristics	Inclusion and exclusion criteria
Study type	• Clinical trials
Participants	• Aged over 18 years with unresectable HCC • Not treated with systemic therapy prior to treatment of ICIs-based regimens • No limitation on the gender, nationality, race or previous medical history
Interventions	• The intervention group included ICIs in the first-line treatment regimen, either as monotherapy or in combination with other therapies
Comparator	• All interventions except first-line ICIs-based therapy • Baseline condition
Outcome measures	• Progression free survival (PFS) (RECIST v1.1 & mRECIST v1.1) • Overall survival (OS) • Disease control rate (DCR) (RECIST v1.1 & mRECIST v1.1) • Objective response rate (ORR) (RECIST v1.1 & mRECIST v1.1) • Treatment-related adverse events (TRAEs) (National cancer institute common terminology criteria for adverse events, v4.0)
Language	• English

### Literature search

Four databases (PubMed, Web of Science, Embase, and clinicaltrials.gov) were searched for eligible studies. The search time was from the inception of the database to 11st August 2022. The main search terms were “hepatocellular carcinoma”, “immune checkpoint inhibitor”, “PD-1”, “PD-L1”, “CTLA-4”, and their synonyms. Abstracts of recent major oncology conferences and included studies' references are also screened. The specific search strategy is shown in Supplementary materials.

### Literature selection

The searched literature was screened using the literature management software Endnote X9. After removing duplicates, two researchers completed screening independently, and the selection included two rounds. In the first round, we reviewed titles and abstracts, and excluded irrelevant literature or literature that did not meet the inclusion criteria. We read the remaining literature in full text in the second round to complete the selection. In case of disagreement, a third and more senior researcher was to make a final judgement.

### Data extraction

Two researchers extracted data separately, with a third researcher making the final judgement in case of any disagreement. The extracted information included the following: (1) basic information of the clinical trial, including authorship, publication date and clinical trial registration number; (2) study design of the clinical trial, including sample size, location, allocation, intervention model, masking, primary purpose, and follow-up time; (3) basic characteristics of included patients, including gender ratio, median age, and basic liver condition; (4) treatments of intervention group including drug and doses; (5) treatment of control group including drug and doses if available; and (6) outcomes of this study, including PFS, OS, ORR, DCR, TRAEs.

### Quality assessment

Eligible studies were assessed by two authors using Cochrane risk of bias (ROB) tool 2.0 for randomized controlled trials (RCT) [24] and Newcastle–Ottawa scale (NOS) for single-arm clinical trials [25]. ROB tool 2.0 judged clinical trials from five perspectives. As long as one of these perspectives was identified as high risk, the study would be evaluated as high risk. NOS took a score value, with a score of 7 or more considered to be of high quality.

### Statistical Analysis

The 'meta' packages in the R language was used to complete this meta-analysis with the frequency methods [26]. The pooled results of continuous variables were shown in standard deviation and 95% confidence interval (CI), and the results of dichotomous variables were shown in OR value and 95% CI [27]. I^2^ was used to evaluate heterogeneity. When I^2^ was smaller than 50% indicating a low level of heterogeneity, a fixed-effect model was used. Otherwise, a random-effect model was used whenever the heterogeneity was considered high. The significance level was set at 0.05, with *P* less than 0.05 indicating a significant difference. Subgroup analysis and sensitivity analysis were used to explain heterogeneity with sufficient data [28].

The 'netmeta' package in the R language was used to perform the network meta-analysis with the frequency methods. The methods for synthesizing results, determining whether to use a random-effect model or a fixed-effect model and evaluating whether there existed a significant difference, were the same as meta-analysis.

Forest plot was done using the 'forest' function in R to visualize the difference in effectiveness among interventions in both meta-analysis and network meta-analysis [27]. Software Stata 15 was used to draw a network of relationships to represent the comparative relationships that existed among interventions. The size of the vertices indicated the sample size included in each intervention, and the thickness of the lines indicated the number of studies included [29].

Using the 'netrank' function in the 'netmeta' package in R to calculate P-scores, we get established all interventions' rank. The P-score depended on the estimating result and confidence interval of the effect value, from 0 to 1, indicating a progressive increase in the efficacy of the included drugs [30].

### Publication bias

Funnel plots were drawn using the 'funnel' function in 'meta' and 'netmeta' package in R to visualize publication bias and then quantified by Egger's test if there were significant asymmetries in the funnel plots [31].

## Results

### Included study

**Fig. 1:**
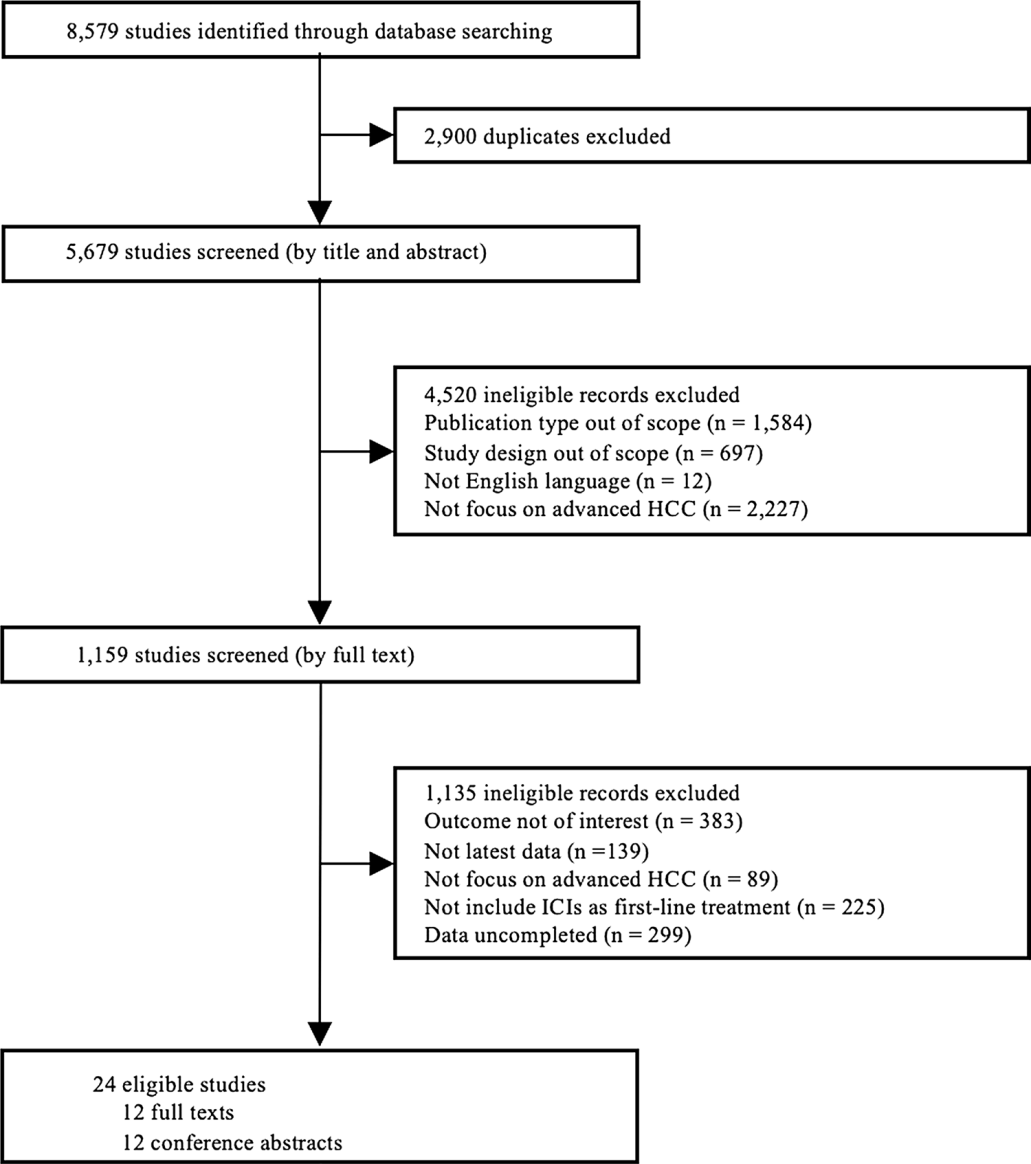
PRISMA flowchart of study selection

Initially, 8579 studies were identified through database searching. Of them, 2900 were excluded because of duplication. The first round of selection of 5679 remaining studies was completed by reviewing titles and abstracts. After the 1st round screening, 4520 studies were excluded because of the following reasons: publication types of 1584 studies were out of scope; study designs of 697 studies were out of scope; 12 studies were not English publications; and 2227 studies did not focus on unresectable HCC. Then, 1159 studies were reviewed by full text in the second round. Of which, 1135 studies were excluded for the following reasons: outcomes of 383 studies were not of interest; 139 studies did not report latest clinical trial data; 89 studies did not focus on unresectable HCC; 225 studies did not take ICIs as first-line treatment; and 299 studies had not completed yet. Finally, 24 studies were included in this review, including 12 full texts and 12 conference abstracts (Fig. 1).

### Study characteristics

Study characteristics of all the included studies were listed in Table 2. A total of 10 studies were multi-center, nine were conducted in mainland China, and of the remaining trials, three were in Japan and one each in the USA and Korea.

**Table 2:** Characteristics of included study

Author, year	Registered number, Acronym	Phase	Region	Study design	Baseline characteristics of study population			
					Age (years) (mean or median)	Male (%)	BCLC stage (%)	Child–Pugh class (%)
Kudo 2021 [32]	NCT01658878, CheckMate 040	1/2	Multi-centre	• Non-Randomized • Parallel Assignment • Open Label	67	NA	4(A), 16(B), 73(C), NR(D)	2(A6), 76(B7), 22(B8)
Yau 2019^a^ [33]	NCT02576509, CheckMate 459	3	Multi-centre	• Randomized • Parallel Assignment • Open Label	64.2	84.9	NA	NA
Finn 2020 [41]	NCT03006926	1	Multi-centre	• NA • Single Group Assignment • Open Label	66.5	81.0	29(B), 71(C)	71(A5), 27(A6), 2(B7)
Finn 2020 [13]	NCT03434379, IMbrave 150	3	Multi-centre	• Randomized • Parallel Assignment • Open Label	Atezo + Bev 64 SOR 66	Atezo + Bev 82 SOR 83	Atezo + Bev 2(A), 15(B), 82(C) SOR 4(A), 16(B), 81(C)	Atezo + Bev 72(A5), 28(A6) SOR 73(A5), 27(A6)
Xu 2021 [42]	NCT03463876 RESCUE	2	Mainland China	• NA • Single Group Assignment • Open Label	1-L 53 2-L 51	1-L 90.0 2-L 88.3	1-L 17.1(B), 82.9(C) 2-L 18.3(B), 81.7(C)	1-L 87.1(A5), 12.9(A6) 2-L 84.2(A5), 15.8(A6)
Kim 2021^a^ [43]	NCT03347292	1b	United States	• Non-Randomized • Sequential Assignment • Open Label	67	NA	41(B), 55(C)	100(A)
Jiao 2021^a^ [44]	NCT04344158	3	Mainland China	• Randomized • Parallel Assignment • Open Label	56	NA	23(B), 77(C)	NA
Kudo 2020^a^ [45]	NCT03418922 Study 117	1b	Japan	• Non-Randomized • Sequential A ssignment • Open Label	NA	NA	NA	NA
Kudo 2020 [46]	NCT03289533 VEGF Liver 100	1	Japan	• Non-Randomized • Parallel Assignment • Open Label	68.5	90.9	40.9(B), 59.1(C)	NA
Lee 2020 [35]	NCT02715531 GO30140	1b	Multi-centre	• Randomized • Parallel Assignment • Open Label	A 62 F1 60 F2 63	A 81 F1 90 F2 83	A 0(A), 10(B), 90(C) F1 0(A), 10(B), 90(C) F2 3(A), 7(B), 90(C)	A 74(A5), 20(A6), 6(B7) F1 72(A5), 28(A6), 0(B7) F2 71(A5), 29(A6), 0(B7)
Li 2021 [52]	NCT03092895	1b/2	Mainland China	• Non-Randomized • Parallel Assignment • Open Label	52	91.2	11.8(B), 85.3(C), 2.9(unknown)	97.1(A), 2.9(B)
Lin 2021^a^ [47]	ChiCTR1900028295	2	Mainland China	• NA • Single arm • Open Label	NA	NA	NA	100(≤ B7)
Ren 2021 [36]	NCT03794440 ORIENT-32	3	Mainland China	• Randomized • Parallel Assignment • Open Label	Sintilimab + IBI305 53 SOR 54	Sintilimab + IBI305 88 SOR 90	Sintilimab + IBI305 15(B), 85(C) SOR 14(B), 86(C)	Sintilimab + IBI305 96(A), 4(B) SOR 95(A), 5(B)
Kelley 2022 50]	NCT03755791 COSMIC-312	3	Multi-centre	• Randomized • Parallel Assignment • Open Label	Cabozantinib + Atezo 64 SOR 64	Cabozantinib + Atezo 83 SOR 86	Cabozantinib + Atezo 32(B) 68(C) SOR 33(B) 67(C)	Cabozantinib + Atezo 100(A) SOR 100(A)
He 2021^a^ [53]	NCT04044313	2	Mainland China	• NA • Single Group Assignment • Open Label	49	91.7	NA	NA
Abou-Alfa 2022^a^ [54]	NCT03298451 HIMALAYA	3	Multi-centre	• Randomized • Parallel Assignment • Open Label	NA	NA	NA	NA
Bai 2021^a^ [48]	NCT04444167	2	Mainland China	• NA • Single Group Assignment • Open Label	52.5	86.7	NA	NA
Verset 2022 [34]	NCT02702414 KEYNOTE-224	2	Multi-centre	• NA • Single Group Assignment • Open Label	68	86	22(B), 78(C)	100(A)
Chen 2022 [51]	NCT04052152	2	Mainland China	• NA • Single Group Assignment • Open Label	56	90.0	25(B), 75(C)	95(A), 5(B)
Hao 2022^a^ [37]	NCT04605796	2	Mainland China	• NA • Single Group Assignment • Open Label	54	88.9	74.1(C)	NA
Meyer 2022^a^ [38]	NCT03468426	1b	Multi-centre	• NA • Single Group Assignment • Open Label	1-L 65 2-L 64	1-L 87 2-L 77	NA	NA
Lim 2022^a^ [39]	NCT02519348	2	Multi-centre	• Randomized • Parallel Assignment • Open Label	NA	NA	NA	NA
Yoo 2022^a^ [49]	NCT04310709 RENOBATE	2	Korea	• NA • Single Group Assignment • Open Label	61 (40–79)	73.8	90.5(C)	NA
Maesaka 2022 [40]	NA	NA	Japan	• NA • Parallel Assignment • Open Label	Atezo + Bev 76 Len 73	Atezo + Bev 76.8 Len 76.3	Atezo + Bev 49.3(A/B), 50.7(C) Len 40.7(A/B), 50.3(C)	Atezo + Bev 97.1(A), 2.9(B) Len 79.5(A), 20.5(B)

Author, year	Study drug(s), n	Comparator	Follow-up time (mo)	Outcome(s) studied				
				PFS median, mo	OS median, mo	ORR (%)	DCR (%)	TRAEs (%)
Kudo 2021 [32]	Nivo (SOR naïve), 25	Nivo (SOR treated), 24	16.3 (12.2–22.5)	1-L 3.4 (1.6–4.1) 2-L 2.2 (1.4–4.2)	1-L 9.8 (3.7–14.3) 2-L 7.4 (2.3–12.1)	1-L 12 2-L 13	1-L 60 2-L 50	Any grade: 1-L 44 2-L 58
Yau 2019^a^ [33]	Nivo, 371	SOR, 372	NA	Nivo 3.68 (3.06–3.88) SOR 14.69 (11.89–17.22)	Nivo 16.39 (13.93–18.37) SOR 14.69 (11.89–17.22)	Nivo 15.4 SOR 7.0	NA	SAEs: Nivo 56.40 SOR 58.95
Finn 2020 [41]	Len + Pembro, 100	NA	10.6 (9.2–11.5)	8.2 (7.4–9.7)	22.0 (20.4-NE)	46	86	Grade ≥ 3 TRAEs: 67
Finn 2020 [13]	Atezo + Bev, 336	SOR, 165	Atezo + Bev 8.9 (7.1–11.1) SOR 8.1 (4.7–10.2)	Atezo + Bev 6.83 (5.75–8.28) SOR 4.27 (3.98–5.55)	Atezo + Bev NE SOR 13.2 (10.4-NE)	Atezo + Bev 27.3 SOR 11.9	Atezo + Bev 73.6 SOR 55.3	Any grade: Atezo + Bev 98.2 SOR 98.7
Xu 2021 [42]	Cam + Apa (1L), 70	Cam + Apa (2L), 120	1-L 16.7 (11.1–18.2) 2-L 14.0 (9.6– 17.0)	1-L 5.7 (5.4 -7.4) 2-L 5.5 (3.7–5.6)	1-L NE 2-L NE	1-L 34.3 2-L 22.5	1-L 77.1 2-L 75.8	1-L Grade ≥ 3: 78.6 2-L Grade ≥ 3: 76.7
Kim 2021^a^ [43]	Reg + Pembro, 29	NA	NA	NA	NA	NA	NA	24
Jiao 2021^a^ [44]	Penpulimab + Anlotinib, 31	NA	11.9 (3.7–17.7)	7.6	NE	31	82.8	Grade ≥ 3: 16.1
Kudo 2020^a^ [45]	Len + Nivo, 24	NA	NA	NA	NA	79.2	NA	NA
Kudo 2020 [46]	Avelumab + axitinib, 22	NA	NA	5.52 (1.91–7.39)	14.05 (7.95-NE)	13.6	68.2	Grade 3–4: 77.2
Lee 2020 [35]	A: Atezo + Bev, 104	F1: Atezo + Bev, 60 F2: Bev, 59	A 6.6 (5.5–8.5) F1 6.6 (5.5–8.5) F2 6·7 (4.2–8.2)	A 7.3 (5.4–9.9) F1 5.6 (3.6–7.4) F2 3.4 (1.9–5.2)	A NE F1 NE F2 NE	A 36 F1 20 F2 17	A 71 F1 67 F2 49	Grade 3–4: A 53 F1 17 F2 5
Li 2021 [52]	Cam + FOLFOX4, 34	NA	11.5 (2.7–22.4)	7.4 (3.9- 9.2)	6 mo 79.4% 12 mo 50.0%	29.4	79.4	Grade ≥ 3 TRAEs: 85.3
Lin 2021^a^ [47]	Anlotinib + toripalimab, 30	NA	NA	NA	NA	25	87.5	Grade 3: 45.5
Ren 2021 [36]	Sintilimab + IBI305, 380	SOR, 191	Sintilimab + IBI305 10.0 (8.5–11.7) SOR 10.0 (8.4–11.7)	Sintilimab + IBI305 4.6 (4.1–5.7) SOR 2.8 (2.7–3.2)	NA	Sintilimab + IBI305 21 SOR 4	Sintilimab + IBI305 4.6 72 SOR 64	Grade ≥ 3: Sintilimab + IBI305 55 SOR 48.1
Kelley 2022 [50]	Atezo + Cabozantinib, 432	SOR, 217, Cabozantinib, 188	15.8 (14.5–17.2)	Cabozantinib + Atezo 6.8 (99% CI 5.6–8.3) SOR 4.2 (99% CI 2.8–7.0)	Cabozantinib + Atezo 15.4 (96% CI 13.7–17.7) SOR 12.2 (96% CI 15.5-NE)	Cabozantinib + Atezo 11 SOR 4	Cabozantinib + Atezo 78 SOR 65	Any grade: 93
He 2021^a^ [53]	Toripalimab + LEN + HAIC, 36	NA	11.2	10.5 (6.21 − 14.79)	NA	63.9	NA	Grade ≥ 3: 72.2
Abou-Alfa 2022^a^ [54]	Tremelimumab + durvalumab, 393	Durvalumab, 389, SOR, 389	STRIDE 16.1 Durvalumab 16.5 SOR 13.3	STRIDE 3.8 (3.7–5.3) Durvalumab 3.7 (3.2–3.8) SOR 4.1 (3.8–5.5)	STRIDE 16.4 (14.2–19.6) Durvalumab 16.6 (14.1–19.1) SOR 13.8 (12.3–16.1)	STRIDE 20.1 D 17.0 S 5.1	NA	Grade ≥ 3: 25.8
Bai 2021^a^ [48]	AK104 + LEN, 30	NA	NA	NA	NA	44.4	77.8	Any grade: 83.3
Verset 2022 [34]	Pembro, 51	NA	27 (range, 23–29)	4 (2–8)	17 (8–23)	16	57	Any grade: 55
Chen 2021^a^ [51]	Sintilimab + anlotinib, 20	NA	12 (range 3–19)	12.2(3.8-NR)	NA	40	95	Any grade: 100
Hao 2022^a^ [37]	Toripalimab + Bev, 54	NA	NA	9.9 (5.5–11.0)	NE	32.7	78.8	Grade ≥ 3: 25.9
Meyer 2022^a^ [38]	Ezabenlimab + BI 836,880, 1-L 30	Ezabenlimab + BI 836,880, 2-L 31	NA	NA	NA	21	85.7	Any grade: 48
Lim 2022^a^ [39]	Durvalumab + Bev, 47	NA	NA	NA	NE	21.3	NA	Any grade: 70.2
Yoo 2022^a^ [49]	Nivo + regorafenib, 42	NA	9.2 (8.5–9.9)	5.5 (1.8–9.1)	NE	31	NA	NA
Maesaka 2022 [40]	Atezo + Bev, 69	LEN, 161	NA	Atezo + Bev 8.8 LEN 5.2	Atezo + Bev NR LEN 20.6	Atezo + Bev 29.7 LEN 30.2	Atezo + Bev 71.9 LEN 82.5	Any grade: 98.5

1-L, first-line; 2-L, second-line; Atezo, atezolizumab; BCLC, Barcelona Clinic Liver Cancer; Bev, bevacizumab; Cam, camrelizumab; FOLFOX4, fluorouracil, leucovorin, and oxaliplatin; LEN, lenvatinib; mo, month; NA, not available; NE, not estimable; Nivo, nivolumab; Pembro, pembrolizumab; REG, regorafenib; SOR, sorafenib; SAEs, serious adverse events; STRIDE, tremelimumab plus durvalumab; TRAEs, treatment-related adverse events

^a^Conference abstract

#### Study design

All clinical trials were open-label and designed for treatment. Among them eight were randomized, five were non-randomized, and the allocation method of the remaining eleven clinical trials was not provided. Six trials were phase I, nine were phase II, and two were phase I/II trials, while the remaining seven clinical trials were phase III.

Thirteen of them were single-arm trials, which took patients’ pre-drug condition as comparator. Three trials compared ICIs-based therapy as first-line and second-line treatment, five selected one kind of TKIs as direct comparators, and three other clinical trials were set up with three different treatment regimens, including a direct comparison among three different ICIs-based therapies, a direct comparison among two ICIs-based therapies and TKIs, and a direct comparison among ICIs-based and two TKIs.

#### Study population

The sample size of included clinical trials ranged from 16 to 1171. Most of clinical trials were predominantly male, the gender ratio ranged from 73.8 to 91.7%. The median age of included population varied from 49 to 76 years. Eight clinical trials reported the HCC aetiology of patients, most of whom were HBV or HCV infection. The majority of patients were class A on the Child–Pugh, and the performance status score on Eastern Cooperative Oncology Group (ECOG) was of 0 or 1.

#### Study drugs

ICIs as first-line treatment in clinical trials included in this study were of three types, including anti-programmed cell death protein 1 (PD-1) antibodies, anti-programmed cell death ligand 1 (PD-L1) antibodies, and anti-cytotoxic T-lymphocyte antigen 4 (CTLA-4) antibodies. Nivolumab and atezolizumab was the most investigated drug of all treatment regimens (n = 4), followed by pembrolizumab (n = 3), toripalimab (n = 3), camrelizumab (n = 2), sintilimab (n = 2), durvalumab (n = 2), AK105 (n = 1), avelumab (n = 1), tremelimumab (n = 1), AK104 (n = 1), and ezabenlimab (n = 1).

Three studies with unresectable HCC patients were treated with monotherapy therapy of ICIs [32–34]. Two studies focused on the efficacy and safety of nivolumab, and the remaining one focused on pembrolizumab. A total of seven studies used combination therapy of ICIs and angiogenesis inhibitory monoclonal antibodies (AI mAbs) as the treatment of intervention group [13, 35–40]. Three studies examined atezolizumab plus bevacizumab, followed by sintilimab plus IBI305 (n = 1), toripalimab plus bevacizumab (n = 1), ezabenlimab plus BI 836,880 (n = 1), and durvalumab plus bevacizumab (n = 1). Eleven eligible studies were about combination therapy of ICIs and TKIs [41–51] involving 9 kinds of ICIs. The remaining three studies chose ICIs with ICIs, chemotherapy, and TKIs plus hepatic arterial infusion chemotherapy (HAIC) as first-line regimens, respectively [52–54].

### Quality assessment

Thirteen clinical trials were single-arm studies, of which the quality was evaluated with NOS from three perspectives. As shown in Table 3, the score of these thirteen studies were all the same indicating a common concern on the comparability because the study design was a single group assignment.

**Table 3:** Quality assessment of single-arm clinical trials

Author, year	Selection	Comparability	Outcome	Score
Finn 2020 [41]	3	0	3	6
Kim 2021* [43]	3	0	3	6
Kudo 2020* [45]	3	0	3	6
Kudo 2020 [46]	3	0	3	6
Li 2021 [52]	3	0	3	6
Lin 2021* [47]	3	0	3	6
He 2021* [53]	3	0	3	6
Bai 2021* [48]	3	0	3	6
Verset 2022 [34]	3	0	3	6
Chen 2022 [51]	3	0	3	6
Hao 2022* [37]	3	0	3	6
Lim 2022* [39]	3	0	3	6
Yoo 2022* [49]	3	0	3	6

Studies marked with asterisk are conference abstracts

**Fig. 2:**
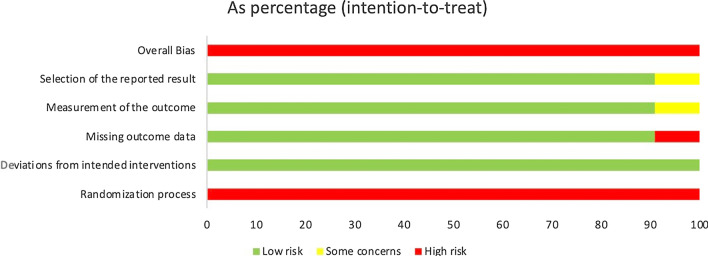
Quality assessment of controlled clinical trials

Eleven controlled clinical trials were involved in the network meta-analysis. Firstly, we evaluated them using the Cochrane ROB Tool (2.0) for RCTs. Considering that all of them were open-label, they were marked high risk in randomization process domains. Thus, all these ten controlled trials were of high risk. The specific results are shown showed in Fig. 2.

### Survival outcomes

#### Pooled results of PFS

Eighteen studies reported median PFS. Two studies compared efficacy of first-line and second-line treatment, and both of them reported a longer median PFS displayed in first-line group [32, 42].

**Fig. 3:**
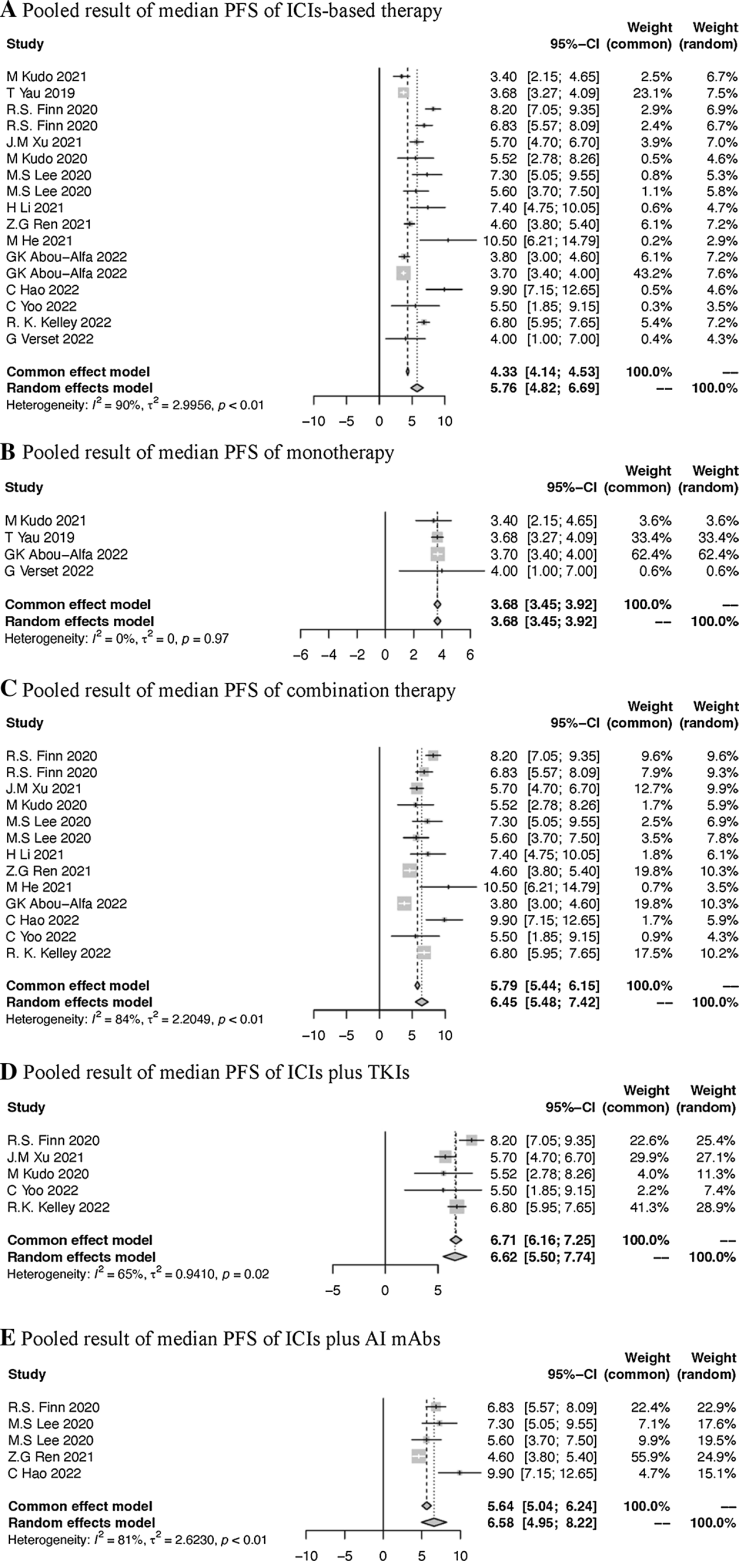
Pooled results of PFS

The pooled result was 5.76 months (95% CI 4.82–6.69) (Fig. 3A), including four monotherapy and 13 combination therapy. The median PFS of monotherapy and combination therapy was 3.68 months (95% CI 3.45–3.92) and 6.45 months (95% CI 5.48–7.42) (Fig. 3B, C) respectively. For subgroups of patients who received ICIs plus TKIs and ICIs plus AI mAbs, the median PFS was 6.62 months (95% CI 5.50–7.74) vs 6.58 months (95% CI 4.95–8.22) (Fig. 3C, D). In the pooled analysis for PFS, all subgroups within the combination therapy group showed a median PFS exceeding the 6 months compared to the monotherapy group.

**Fig. 4:**
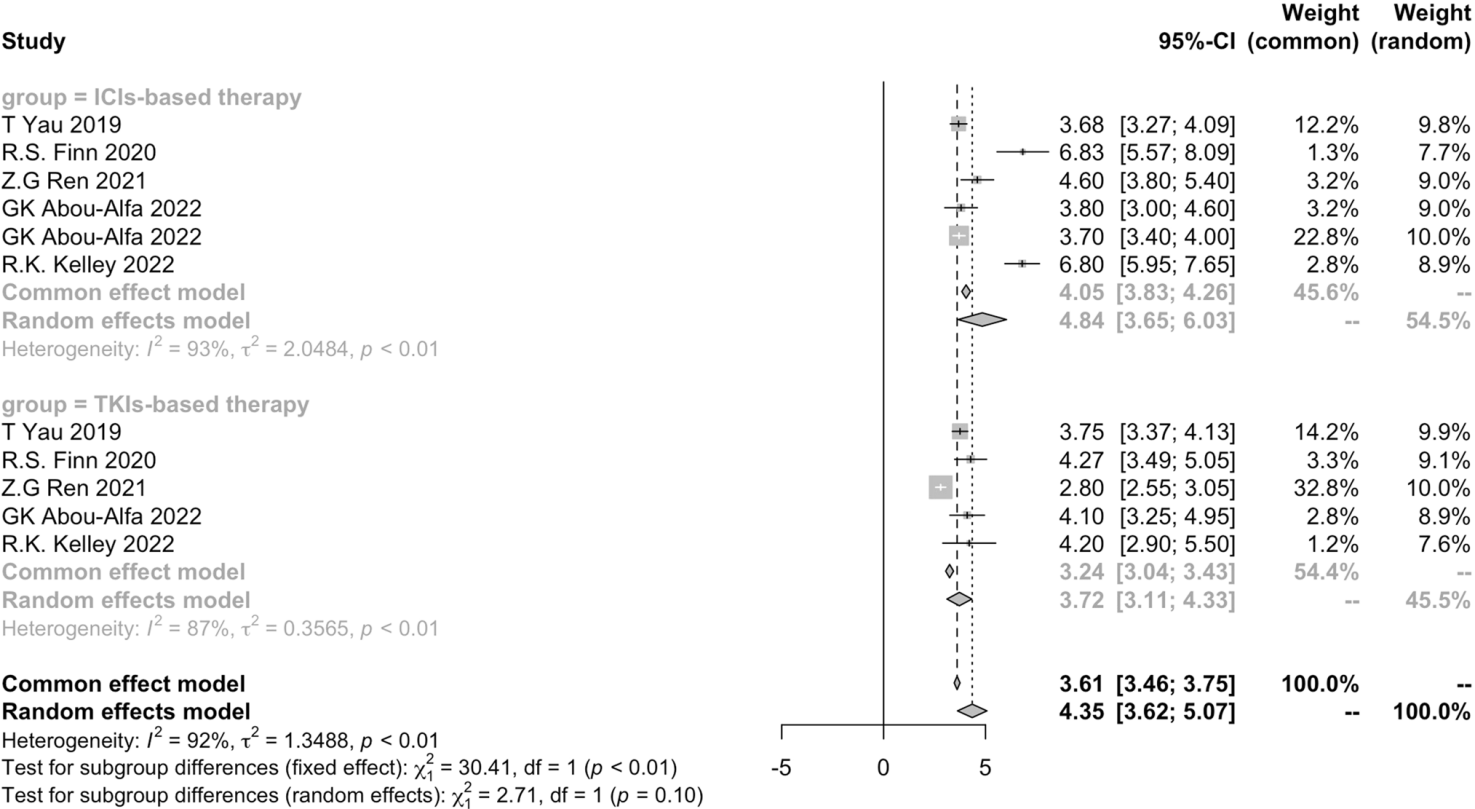
Pooled results of PFS of ICIs vs TKIs

As showed in Fig. 4, six cohorts from five studies compared ICIs-based immunotherapy with TKIs, and all of them chose sorafenib as the control group [13, 33, 36, 50, 54]. The pooled results showed that in these four randomized control trials, the median PFS of ICIs-based group was 4.84 months (95% CI 3.65–6.03), and that of TKIs-based therapy was 3.72 months (95% CI 3.11–4.33). The *P* value between two regimens was 0.10, which meant no significant difference existed. It implied that the efficacy of ICIs-based therapy and TKIs-based was the same in prolonging PFS.

#### Pooled results of OS

Only six clinical trials reported median OS. Two studies compared the efficacy of first-line and two-line treatment. One reported median OS of first-line group was 9.8 months (95% CI 3.7–14.3), and 7.4 months (95% CI 2.3–12.1) of second-line group indicating that first-line ICIs-based treatment could give a longer OS for HCC patients [32]. However, another study reported that the OS was similar between two groups in patients who had a complete/partial response, stable disease, or progressive disease. In addition, as summarized by the authors, unresectable HCC patients could benefit in OS from the use of ICIs-based treatment regardless first-line or second-line [42].

**Fig. 5:**
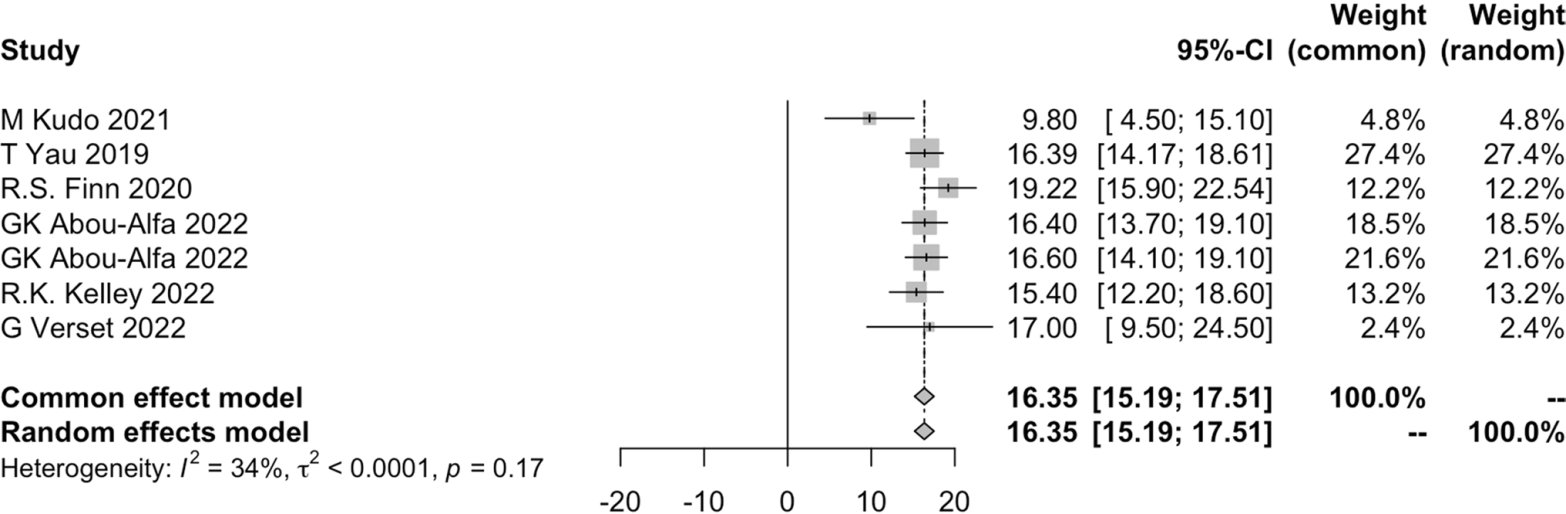
Pooled results of median OS

The pooled result of five studies was 16.35 months (95% CI 15.19–17.51) (Fig. 5). Due to the follow-up time was not long enough, it was immature to include the median OS from the other studies.

**Fig. 6:**
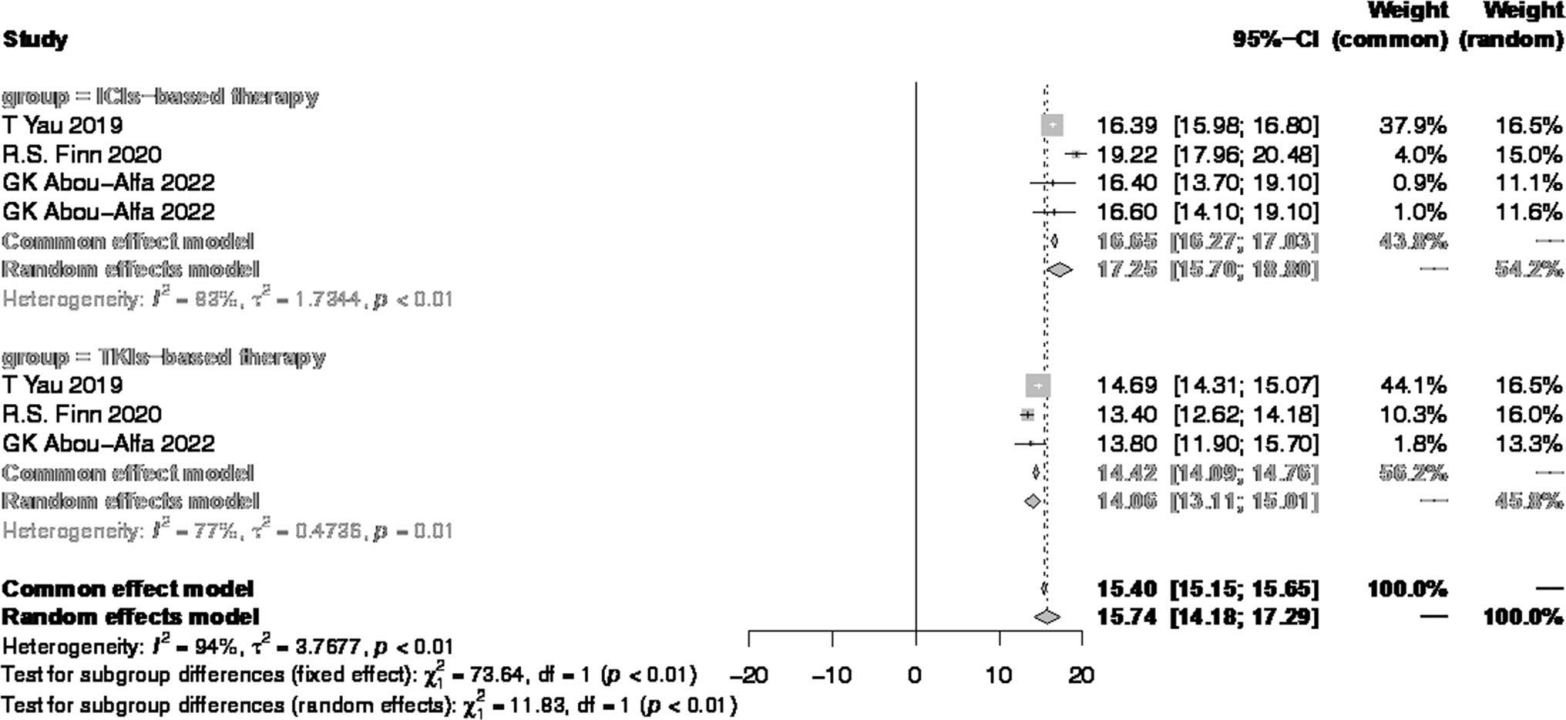
Pooled results of OS of ICIs vs TKIs

Three studies reported comparing ICIs-based treatment and TKIs [13, 33, 54]. As Fig. 6 showed, the median OS was 17.40 months (95% CI 15.45–19.36) vs 14.06 months (95% CI 13.11–15.01), and the p-value was smaller than 0.01. The results illustrated unresectable HCC patients could benefit in survival by choosing ICIs as their first-line treatment.

### Best response rate

As shown in Supplementary Table S1, 25 groups reported ORR of the trials while 19 reported DCR of the patients. ORR and DCR were 25.1% (95% CI 20.8%-29.5%) and 75.2% (95% CI 70.3%-80.2%) as evaluated with response evaluation criteria in solid tumours (RECIST) v1.1. When measured with modified RECIST (mRECIST) v1.1, the pooled outcomes were 40.2% (95% CI 31.7%-48.6%) with 75.2% (95% CI 68.3%-82.1%).

We also calculated the pooled results for subgroups in Supplementary Table S1. When measured with RECIST v1.1, ORR and DCR of monotherapy was 16.1% (95% CI 13.7–18.5%) and 54.1% (95% CI 45.8–62.5%), and for the combination group, ORR and DCR were 27.8% (95% CI 22.8–32.8%) and 78.2% (95% CI 74.3–82.2%) respectively. When measured by mRECIST v1.1, because of the lack of results of monotherapy, we only got pooled results of combination therapy and its subgroups. The pooled result of ORR was 41.9% (95% CI 33.7–50.2%) for patients who received combination therapy, and DCR was 77.5% (95% CI 72.1–83.0%).

The pooled results of response rate revealed that ICIs plus TKIs group was more beneficial to unresectable HCC patients than ICIs plus AI mAbs regimens.

### Pooled result of TRAEs

**Fig. 7:**
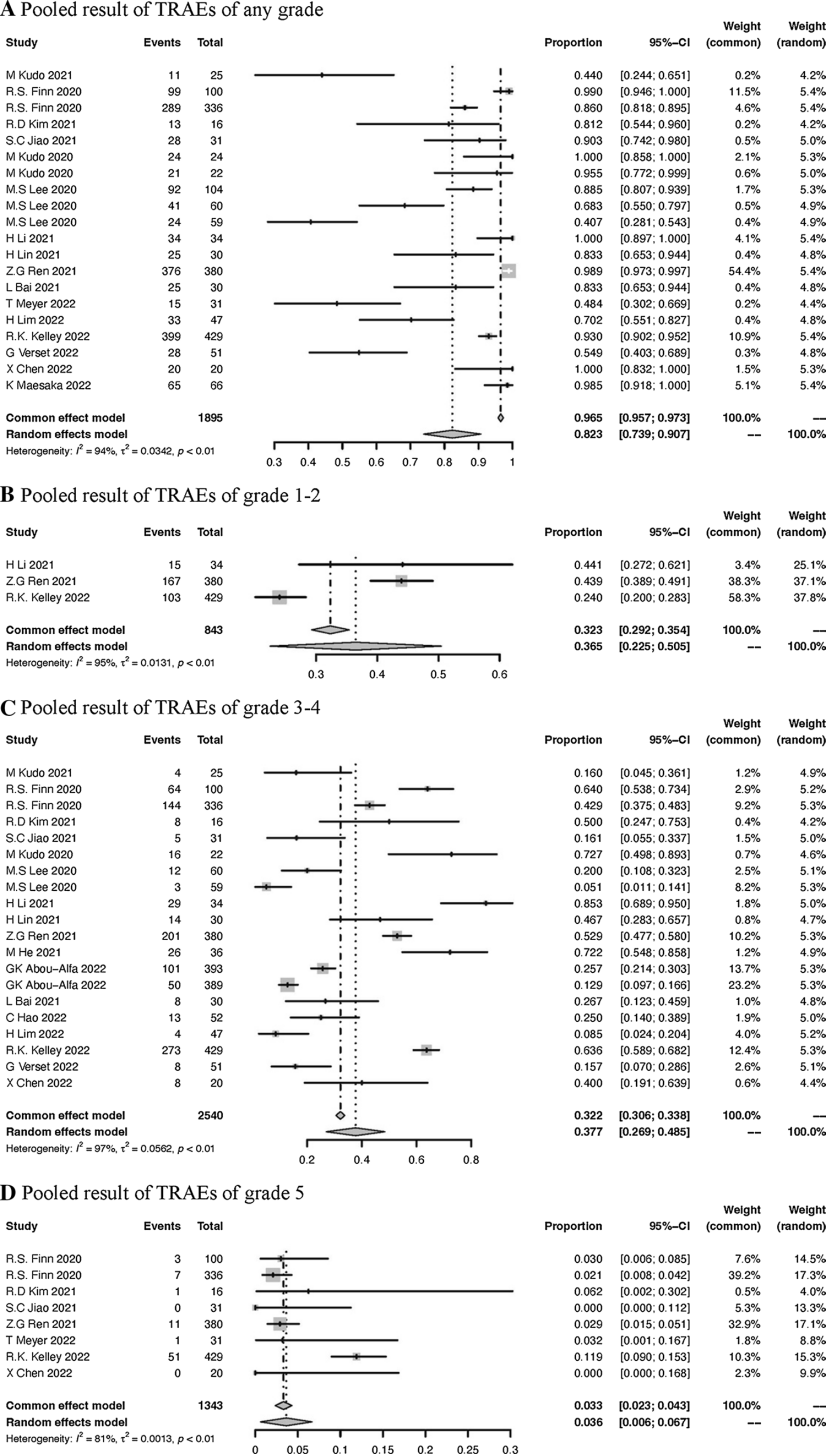
Pooled results of TRAE

All 24 studies reported the rate of TRAEs. The incidence of any adverse events was 82.3% (95% CI 73.9%-90.7%) (Fig. 7A). Calculated according to different grades of adverse events, including grade1-2, grade3-4 and grade 5, the pooled results were 36.5% (95% CI 22.5–50.5%), 37.7% (95% CI 26.9–48.5%) and 3.6% (95% CI 0.6–6.7%) respectively (Fig. 7B, C, D). Moreover, we summarized the incidence of each adverse event in Supplementary Table S2. It was easy to discover that the most common adverse events differed in different grades. The most common adverse event of any grade was dysphonia, while the most common adverse in grade 1–2 and grade 3–4 were proteinuria and hypertension, respectively.

### Network meta-analysis

#### Network meta-analysis of ORR

**Fig. 8:**
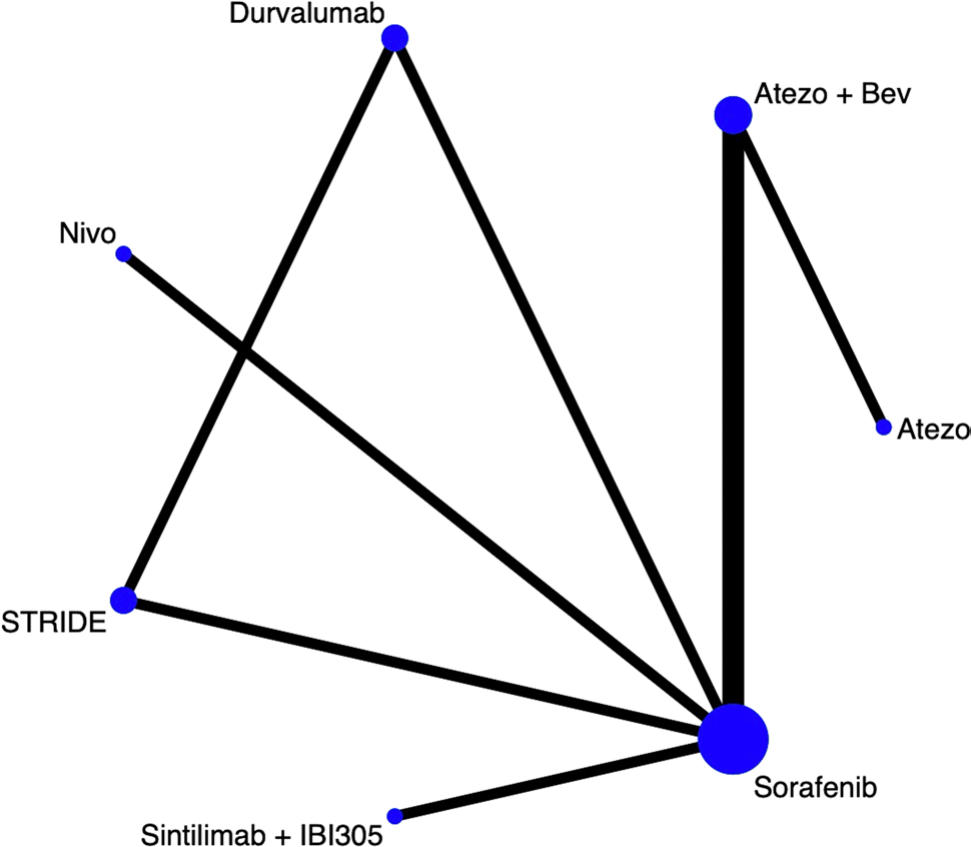
Network plot of ORR

**Fig. 9:**
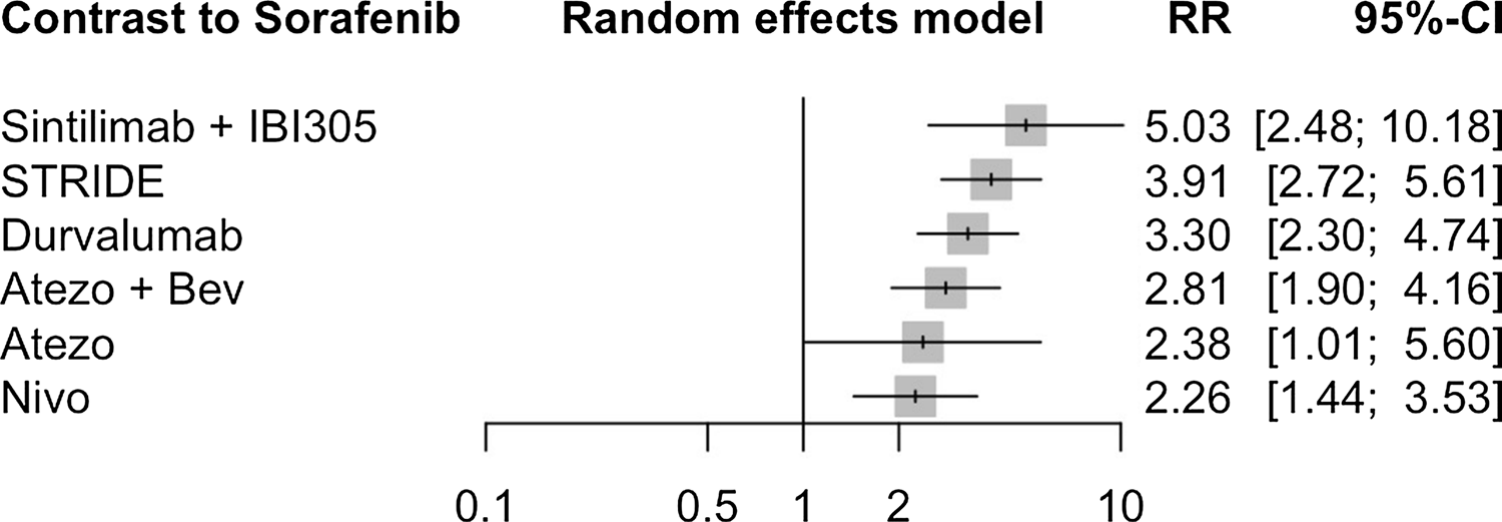
Forest plot for ORR compared to sorafenib

Six clinical trials involved 3,741 patients had reported ORR. Based on these seven treatment options, we set up a network plot displayed as shown in Fig. 8. It clearly indicates that, compared to sorafenib as first-line treatment, all ICIs-based treatment regimens showed a significant improvement in ORR (Fig. 9).

We also did further analysis to indirectly compare the results between every two regimens (Table 4). It showed the same conclusion as meta-analysis that using ICIs-based treatment as first-line therapy could benefit patients more than sorafenib.

**Table 4:** Results of network meta-analysis

Atezo	0.26 (0.19; 0.35)	NA	NA	0.29 (0.21; 0.42)	0.90 (0.65; 1.26)	NA
0.85 (0.40; 1.81)	Atezo + Bev	NA	NA	1.14 (0.98; 1.32)	3.51 (3.21; 3.84)	NA
0.72 (0.29; 1.83)	0.68 (0.39; 1.22)	Durvalumab	NA	NA	NA	NA
1.06 (0.40; 2.77)	1.25 (0.69; 2.26)	1.46 (0.82; 2.60)	Nivo	NA	NA	NA
0.47 (0.16; 1.43)	0.56 (0.25; 1.25)	0.66 (0.30; 1.45)	0.45 (0.19; 1.03)	Sintilimab + IBI305	3.09 (2.73; 3.50)	NA
2.38 (1.01; 5.60)	2.81 (1.90; 4.16)	3.30 (2.30; 4.74)	2.26 (1.44; 3.53)	5.03 (2.48; 10.18)	Sorafenib	NA
0.61 (0.24; 1.54)	0.72 (0.42; 1.23)	0.84 (0.64; 1.11)	0.58 (0.32; 1.02)	1.29 (0.58; 2.84)	0.26 (0.18; 0.37)	STRIDE

Atezo, atezolizumab; Bev, bevacizumab; NA, not available; Nivo, nivolumab; STRIDE, tremelimumab plus durvalumab

#### Network meta-analysis of DCR

**Fig. 10:**
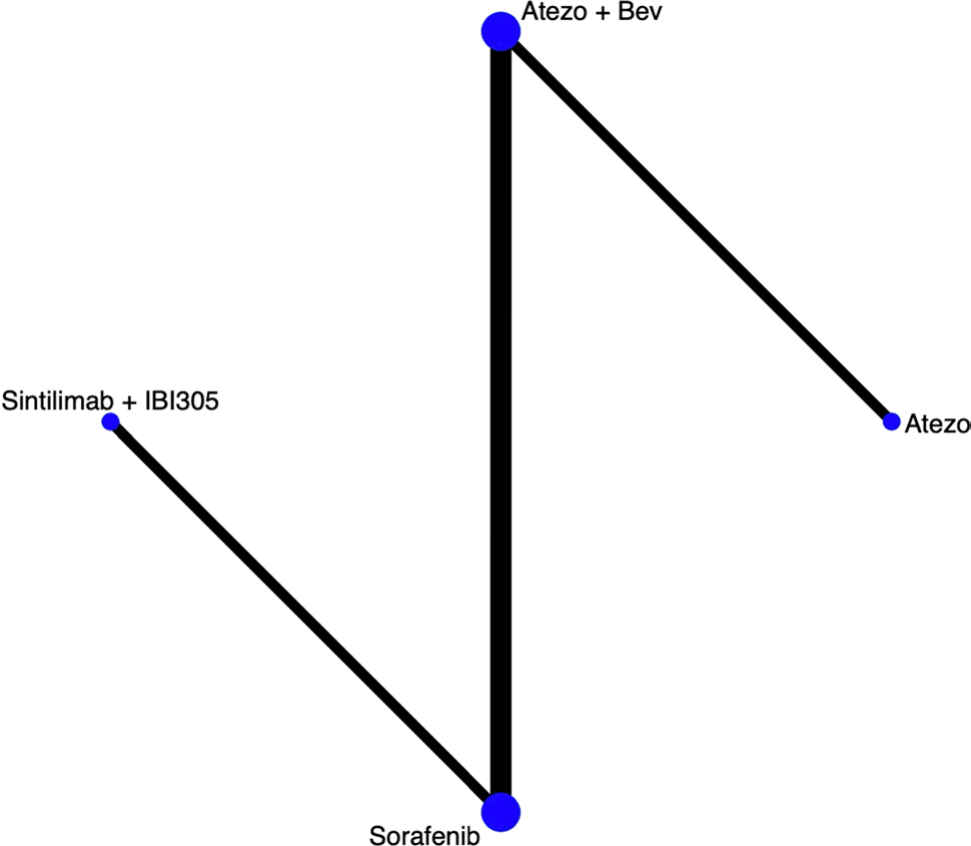
Network plot of DCR

**Fig. 11:**
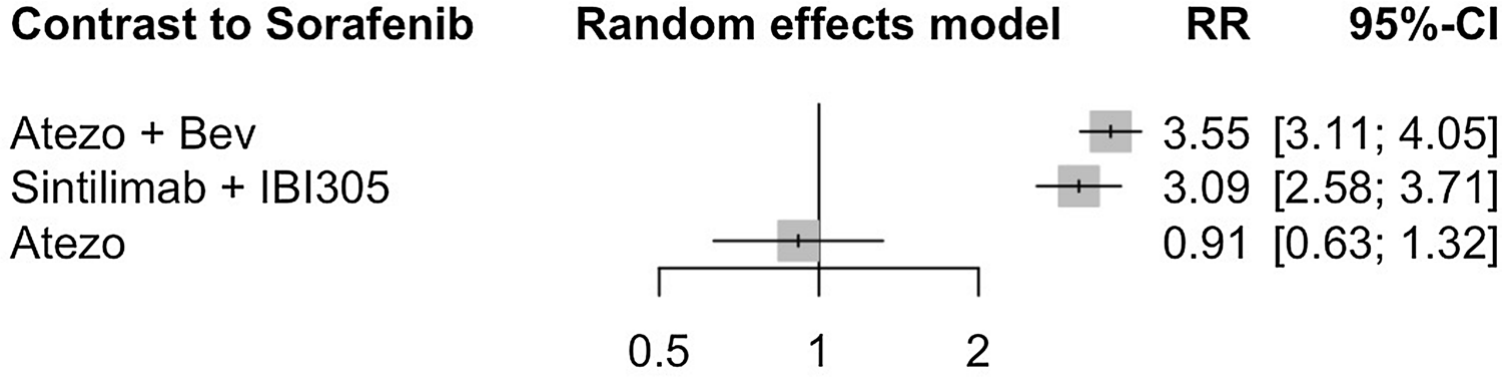
Forest plot for DCR compared to sorafenib

Different from ORR, only four clinical trials reported DCR. As seen in Fig. 10, four treatment options from three clinical studies were involved in this analysis of DCR. After using random effect model to evaluate the difference of DCR among four therapies, as shown in Fig. 11, combination therapy including atezolizumab plus bevacizumab 3.55 (95% CI 3.11–4.05) and sintilimab plus IBI305 3.09 (95% CI 2.58–3.71) both showed significant differences on DCR compared to sorafenib, while the result atezolizumab monotherapy was 0.91 (95% CI 0.63–1.32). The indirect results revealed that combination therapy would be a better choice than monotherapy.

### Publication bias

Publication bias existed in the analysis of median PFS via funnel plot shown in Supplementary Fig S1 and egger’s test shown in Supplementary Table S3, while no obvious publication bias of median OS was identified. For best responses, as shown in Supplementary Fig S2 and Supplementary Table S4, publication bias was observed in the pooled results of CR and ORR, and when measured by RECIST v1.1, the result of PR also showed a publication bias, while SD, PD and DCR did not show any significant publication bias as measured with funnel plots and Egger’s test.

## Discussion

This systematic review, meta-analysis and network meta-analysis included 24 clinical trials, including 13 single-arms and 11 controlled trials, to evaluate the efficacy and safety of ICIs-based therapy as first-line treatment option for unresectable HCC patients. The results of this research indicated that the use of ICIs-based therapy could benefit unresectable HCC patients in both efficacy and safety. Our research findings also raise some issues that are worth discussing as below.

The NCCN guideline currently recommended 2 first-line treatment options including ICIs for specific unresectable HCC patients who provided category 1 evidence, while the CSCO guideline recommended 4 options. They were recommended because of the good efficacy and safety shown in clinical trials. Now, in this research, we have found that other ICIs-based therapies would also be promising as first-line treatments for patients. For example, sintilimab plus IBI305 are new drugs developed in recent years, of which the efficacy as first-line treatment exceeds all existing regimens underscoring the potential role of this combination therapy.

Before the clinical use of ICIs-based treatment, patients with unresectable HCC mostly accepted sorafenib as first-line therapy, which demonstrated a median OS of 11.4 months (95% CI 8.46–14.47) and median PFS of 4.17 months (95% CI 3.08–5.25) [55]. As a pervious review concluded, the potentiality of ICIs-based therapy brought big hope for both patients and researchers, and quite a lot clinical trials worked on different regimens based on their biological rationale [56]. We verified the great potential of ICIs-based in this study: compared with TKI therapy, ICIs-based therapy had significantly better efficacy in prolonging patients’ survival. Moreover, the combination therapy including TKIs and AI mAbs all demonstrated good efficacy. The anti-angiogenic effects of TKIs and AI mAbs could act synergistically with ICIs to destruct the vasculature of malignant tumours and thereby inhibit their growth [57, 58]. Further analysis revealed that different kinds of therapies had their own advantages: sintilimab plus IBI305 showed the best efficacy in ORR compared with other ICIs-based therapy, while atezolizumab plus bevacizumab performed better in terms of DCR. In general, the addition of ICIs as first-line treatment had a favourable impact on unresectable HCC patient survival. As another review suggested, positive outcomes for the use of ICIs-based therapies showed great potentials for developing more drugs against unresectable HCC [59].

Our research found that all different cohorts reported the rate of adverse events after taking ICIs-based treatment with dysphonia being most commonly reported across all grades of adverse events. For TRAEs of grade 1–2, the increased incidence of raised ALT and proteinuria suggests that clinicians need to pay attention to the patient's liver and kidney function during treatment. For TRAEs of grade 3–4, commonly reported hypertension and pyrexia warrant continuous monitoring of patients’ physical condition and adjustments in the treatment whenever necessary. Additionally, it is suggested that researchers could report the incidence of immune-related adverse events for a more detailed analysis.

Regarding clinical research design, it is worth noting that most clinical trials were open label, which might pertain high risks of bias leading to low reliability of the results. Also, 18 of 24 were single-arm trials or could not be used to build a meta-analysis, which meant no sufficient evidence to support the results to fully evaluate the performance of different treatment regimens. In addition, the follow-up time in some included studies was not long enough, resulting in data immature for calculating the median OS. As most existing studies were non-comparative, open-label, and small, it could lead to high heterogeneity and publication bias. Therefore, randomized controlled trials with masking and high-quality are urgently needed. What’s more, more comparative studies involving head-to-head comparisons are required to build a strong and reliable network to build a more robust conclusion about the advantages of different regimens. Since that the stage of liver function of most included participants were Child Pugh A, the limitation of participants may lead we missed the good efficacy for more HCC patients. just as other researchers did to verify the efficacy of metronomic capecitabine, we suggested do similar research for Child Pugh B patients [60, 61].

To our knowledge, this is the first study combined systematic review, meta-analysis and network meta-analysis about clinical trials of ICIs-based therapy as first-line treatment for unresectable HCC patients. However, there are some limitations worth attention. Firstly, because of the lack of aetiological data, we could not do further subgroup analysis to evaluate the efficacy and safety of ICIs-based therapy for patients of different conditions. Secondly, the quality of included clinical trials was evaluated as poor or fair because of the study design. While it is reasonable considering the urgent needs at clinical, it may lead to some bias in outcomes, especially in the long-term evaluation of OS. Future studies with the improvement of research design will be required to provide more extensive evidence of ICIs for HCC. Thirdly, this study only included clinical trials. It should be under consideration to include relevant real-world data to conduct a further and more reliable evaluation.

## Conclusions

This study showed that ICIs-based therapy could benefit unresectable HCC patients in survival and tumour response rates with manageable adverse events. Furthermore, the combination regimens of atezolizumab plus bevacizumab and sintilimab plus IBI305 presented good outcomes in the evaluation, implying that combination therapy could become a new trend in clinical practice for unresectable HCC patients. However, due to the shortage of study design for existing clinical trials, more comparable and high-quality studies of ICIs-based regimens as first-line treatment are needed.

## Supplementary Information

Additional file 1 (DOCX 715 KB)

## Data Availability

All data we used in this work can be found in the references.

## Abbreviations

HCC: Hepatocellular carcinoma
ICI: Immune checkpoint inhibitors
HBV: Hepatitis B virus
HCV: Hepatitis C virus
TACE: Trans-arterial chemoembolization
TKI: Tyrosine kinase inhibitor
PFS: Progression-free survival
OS: Overall survival
ORR: Objective response rate
DCR: Disease control rate
TRAE: Treatment-rated adverse event
RCT: Randomized controlled trial
NOS: Newcastle–Ottawa scale
ROB: Risk of bias
ECOG: Eastern Cooperative Oncology Group
CI: Confidence interval
AI mAbs: Angiogenesis inhibitory monoclonal antibodies
PD-1: Programmed cell death protein 1
PD-L1: Anti-programmed cell death ligand 1
CTLA-4: Cytotoxic T-lymphocyte antigen 4
HAIC: Hepatic arterial infusion chemotherapy
RECIST: Response evaluation criteria in solid tumours
mRECIST: Modified RECIST
1-L: First-line
2-L: Second-line
Atezo: Atezolizumab
BCLC: Barcelona Clinic Liver Cancer
Bev: Bevacizumab
Cam: Camrelizumab
FOLFOX4: Fluorouracil, leucovorin, and oxaliplatin
LEN: Lenvatinib
Mo: Month
NA: Not available
NE: Not estimable
Nivo: Nivolumab
Pembro: Pembrolizumab
REG: Regorafenib
SOR: Sorafenib
SAEs: Serious adverse events
STRIDE: Tremelimumab plus durvalumab
CR: Complete response
PD: Progressive disease
PR: Partial response
SD: Stable disease
ALT: Alanine aminotransferase
AST: Aspartate aminotransferase
