# Supplementary material for: Efficacy and safety of monotherapy and combination therapy of immune checkpoint inhibitors as first-line treatment for unresectable hepatocellular carcinoma: a systematic review, meta-analysis and network meta-analysis
Source: Discov Oncol. 2022 Sep 28;13:95. doi: 10.1007/s12672-022-00559-1 (PMC9519826; doi:10.1007/s12672-022-00559-1)
Supplement: Supplementary file 1 — Additional file 1 (DOCX 715 KB) [file 12672_2022_559_MOESM1_ESM.docx]

**Supplementary material**

***Efficacy and safety of monotherapy and combination therapy of immune checkpoint inhibitors as first-line treatment for unresectable hepatocellular carcinoma: a systematic review and network meta-analysis***

**Supplementary 1. Search strategies**

1. PubMed

((liver[Title/Abstract] OR hepat*[Title/Abstract]) AND (neoplas*[Title/Abstract] OR cancer*[Title/Abstract] OR tumor*[Title/Abstract] OR tumour*[Title/Abstract] OR carcinoma*[Title/Abstract] OR oncolog*[Title/Abstract] OR malign*[Title/Abstract])) AND ((((Atezolizumab[Title/Abstract] OR Tecentriq[Title/Abstract] OR rg7446[Title/Abstract] OR mpdl3280A[Title/Abstract]) AND (Bevacizumab[Title/Abstract] OR Avastin[Title/Abstract] OR Mvasi[Title/Abstract] OR Zirabev[Title/Abstract])) OR (Pembrolizumab[Title/Abstract] OR MK-3475[Title/Abstract] OR lambrolizumab[Title/Abstract] OR Keytruda[Title/Abstract] OR Nivolumab[Title/Abstract] OR opdivo[Title/Abstract] OR bms-936558[Title/Abstract] OR mdx-1106[Title/Abstract] OR Camrelizumab[Title/Abstract] OR SHR-1210[Title/Abstract] OR AiRuiKa[Title/Abstract] OR HR-301210[Title/Abstract])) OR (PD-1 antibody[Title/Abstract] OR PD-L1 antibody[Title/Abstract] OR CTLA-4 antibody[Title/Abstract] OR PD-1 inhibitor[Title/Abstract] OR PD-L1 inhibitor[Title/Abstract] OR CTLA-4 inhibitor[Title/Abstract]))

1. Web of Science

1 ((TS=((liver OR hepat*))) OR TI=((liver OR hepat*))) OR AB=((liver OR hepat*))

2 ((TS=(neoplas* OR cancer* OR tumor* OR tumour* OR carcinoma* OR oncolog* OR malign*)) OR TI=(neoplas* OR cancer* OR tumor* OR tumour* OR carcinoma* OR oncolog* OR malign*)) OR AB=(neoplas* OR cancer* OR tumor* OR tumour* OR carcinoma* OR oncolog* OR malign*)

3 ((TS=(Atezolizumab or Tecentriq or rg7446 or mpdl3280A)) OR TI=(Atezolizumab or Tecentriq or rg7446 or mpdl3280A)) OR AB=(Atezolizumab or Tecentriq or rg7446 or mpdl3280A)

4 ((TS=(Bevacizumab or Avastin or Mvasi or Zirabev)) OR TI=(Bevacizumab or Avastin or Mvasi or Zirabev)) OR AB=(Bevacizumab or Avastin or Mvasi or Zirabev)

5 (#3) AND #4

6 ((TS=(Pembrolizumab or MK-3475 or lambrolizumab or Keytruda or Nivolumab or opdivo or bms-936558 or mdx-1106 or Camrelizumab or SHR-1210 or AiRuiKa or HR-301210)) OR TI=(Pembrolizumab or MK-3475 or lambrolizumab or Keytruda or Nivolumab or opdivo or bms-936558 or mdx-1106 or Camrelizumab or SHR-1210 or AiRuiKa or HR-301210)) OR AB=(Pembrolizumab or MK-3475 or lambrolizumab or Keytruda or Nivolumab or opdivo or bms-936558 or mdx-1106 or Camrelizumab or SHR-1210 or AiRuiKa or HR-301210)

7 ((TS=(PD-1 antibody or PD-L1 antibody or CTLA-4 antibody or PD-1 inhibitor or PD-L1 inhibitor or CTLA-4 inhibitor)) OR TI=(PD-1 antibody or PD-L1 antibody or CTLA-4 antibody or PD-1 inhibitor or PD-L1 inhibitor or CTLA-4 inhibitor)) OR AB=(PD-1 antibody or PD-L1 antibody or CTLA-4 antibody or PD-1 inhibitor or PD-L1 inhibitor or CTLA-4 inhibitor)

8 ((#5) OR #6) OR #7

9 ((#1) AND #2) AND #8

10 (#9) AND LA=(English)

1. Embase

#1 'liver':ab,ti

#2 'hepat*':ab,ti

#3 #1 OR #2

#4 'neoplas*':ab,ti

#5 'cancer*':ab,ti

#6 'tumor*':ab,ti

#7 'tumour*':ab,ti

#8 'carcinoma*':ab,ti

#9 'oncolog*':ab,ti

#10 'malign*':ab,ti

#11 #4 OR #5 OR #6 OR #7 OR #8 OR #9 OR #10

#12 'atezolizumab':ab,ti

#13 'tecentriq':ab,ti

#14 'rg7446':ab,ti

#15 'mpdl3280a':ab,ti

#16 #12 OR #13 OR #14 OR #15

#17 'bevacizumab':ab,ti

#18 'avastin':ab,ti

#19 'mvasi':ab,ti

#20 'zirabev':ab,ti

#21 #17 OR #18 OR #19 OR #20

#22 #16 AND #21

#23 'pembrolizumab':ab,ti

#24 'mk-3475':ab,ti

#25 'lambrolizumab':ab,ti

#26 'keytruda':ab,ti

#27 'nivolumab':ab,ti

#28 'opdivo':ab,ti

#29 'bms-936558':ab,ti

#30 'mdx-1106':ab,ti

#31 'camrelizumab':ab,ti

#32 'shr-1210':ab,ti

#33 'airuika':ab,ti

#34 'hr-301210':ab,ti

#35 #23 OR #24 OR #25 OR #26 OR #27 OR #28 OR #29 OR #30 OR #31 OR #32 OR #33 OR #34

#36 'pd-1 antibody':ab,ti

#37 'pd-l1 antibody':ab,ti

#38 'ctla-4 antibody':ab,ti

#39 'pd-1 inhibitor':ab,ti

#40 'pd-l1 inhibitor':ab,ti

#41 'ctla-4 inhibitor':ab,ti

#42 #36 OR #37 OR #38 OR #39 OR #40 OR #41

#43 #3 AND #11

#44 #22 OR #35 OR #42

#45 #43 AND #44

1. Clinical trials.gov

Condition or disease = Hepatocellular Carcinoma

Intervention/treatment = Atezolizumab plus Bevacizumab or Pembrolizumab or Nivolumab or Camrelizumab or PD-1 antibody or PD-L1 antibody or CTLA-4 antibody or PD-1 inhibitor or PD-L1 inhibitor or CTLA-4 inhibitor

| **Supplementary Table 1. Best response** | | | | | | |  |
| --- | --- | --- | --- | --- | --- | --- | --- |
|  | RECIST v1.1 | | | mRECIST v1.1 | | |  |
| ICIs-based therapy | No. of studies | Rate (95% CI) | I^2^ (%) | No. of studies | Rate (95% CI) | I^2^ (%) | |
| ORR | 25 | 25.1% (20.8%-29.5%) | 83 | 15 | 40.2% (31.7%-48.6%) | 87 | |
| CR | 18 | 2.2% (0.8%-3.5%) | 73 | 13 | 7.9% (5.0%-10.8%) | 81 | |
| PR | 18 | 21.1% (17.1%-25.1%) | 79 | 13 | 28.2% (22.0%-34.4%) | 73 | |
| SD | 16 | 47.0% (41.8%-53.3%) | 83 | 11 | 38.7% (33.7%-43.7%) | 66 | |
| PD | 16 | 21.6% (16.8%-26.4%) | 81 | 11 | 21.5% (15.2%-27.9%) | 85 | |
| DCR | 19 | 75.2% (70.3%-80.2%) | 75 | 11 | 75.2% (68.3%-82.1%) | 80 | |
|  | RECIST v1.1 |  |  | mRECIST v1.1 |  |  | |
| Monotherapy | No. of studies | Rate (95% CI) | I^2^ (%) | No. of studies | Rate (95% CI) | I^2^ (%) | |
| ORR | 5 | 16.1% (13.7%-18.5%) | 0 | NA | NA | NA | |
| CR | 4 | 2.2% (0%-4.9%) | 59 | NA | NA | NA | |
| PR | 4 | 12.3% (9.4%-15.1%) | 0 | NA | NA | NA | |
| SD | 3 | 38.3% (29.8%-46.9%) | 6 | NA | NA | NA | |
| PD | 3 | 35.9% (27.9%-43.9%) | 0 | NA | NA | NA | |
| DCR | 3 | 54.1% (45.8%-62.5%) | 0 | NA | NA | NA | |
|  | RECIST v1.1 |  |  | mRECIST v1.1 |  |  | |
| Combination therapy | No. of studies | Rate (95% CI) | I^2^ (%) | No. of studies | Rate (95% CI) | I^2^ (%) | |
| ORR | 20 | 27.8% (22.8%-32.8%) | 85 | 14 | 41.9% (33.7%-50.2%) | 87 | |
| CR | 14 | 2.7% (1.0%-4.4%) | 79 | 12 | 8.3% (5.2%-11.4%) | 83 | |
| PR | 14 | 23.7% (19.3%-28.2%) | 81 | 12 | 29.5% (23.8%-35.1%) | 67 | |
| SD | 13 | 48.5% (42.8%-54.1%) | 85 | 10 | 39.3% (34.0%-44.7%) | 67 | |
| PD | 13 | 19.0% (14.6%-23.5%) | 79 | 10 | 19.7% (13.9%-25.5%) | 84 | |
| DCR | 16 | 78.2% (74.3%-82.2%) | 65 | 10 | 77.5% (72.1%-83.0%) | 72 | |
|  | RECIST v1.1 |  |  | mRECIST v1.1 |  |  | |
| ICIs plus TKIs | No. of studies | Rate (95% CI) | I^2^ (%) | No. of studies | Rate (95% CI) | I^2^ (%) | |
| ORR | 9 | 27.3% (19.0%-35.7%) | 86 | 7 | 46.5% (33.9%-59.1%) | 73 | |
| CR | 7 | 0.3% (0%-0.9%) | 0 | 6 | 7.0% (3.9%-10.1%) | 0 | |
| PR | 7 | 24.9% (15.5%-34.4%) | 87 | 6 | 32.7% (22.7%-42.7%) | 60 | |
| SD | 6 | 53.7% (43.4%-64.0%) | 83 | 5 | 39.8% (28.5%-51.0%) | 61 | |
| PD | 6 | 12.5% (7.3%-17.7%) | 59 | 5 | 11.6% (4.7%-18.5%) | 52 | |
| DCR | 8 | 83.1% (77.7%-88.5%) | 59 | 5 | 84.5% (76.5%-92.4%) | 55 | |
|  | RECIST v1.1 |  |  | mRECIST v1.1 |  |  | |
| ICIs plus AI mAbs | No. of studies | Rate (95% CI) | I^2^ (%) | No. of studies | Rate (95% CI) | I^2^ (%) | |
| ORR | 8 | 26.4% (21.9%-30.8%) | 56 | 6 | 33.4% (25.8%-41.0%) | 78 | |
| CR | 6 | 4.0% (0.6%-7.3%） | 89 | 5 | 8.4% (3.0%-13.7%) | 92 | |
| PR | 6 | 22.1% (19.5%-24.7%) | 0 | 5 | 23.8% (21.1%-26.6%) | 0 | |
| SD | 6 | 44.6% (39.3%-49.9%) | 58 | 5 | 39.3% (32.5%-46.1%) | 76 | |
| PD | 6 | 23.9% (20.0%-27.8%) | 39 | 5 | 26.2% (23.4%-29.0%) | 0 | |
| DCR | 7 | 73.2% (70.5%-75.9%) | 10 | 5 | 72.6% (69.7%-75.4%) | 0 | |

Abbreviations: CR, complete response; NA, not available; PD, progressive disease; PR, partial response; SD, stable disease.

| **Supplementary Table 2. Adverse events of first-line ICIs-based treatment** | | | | | | | | | |
| --- | --- | --- | --- | --- | --- | --- | --- | --- | --- |
| Adverse events | Any grade |  |  | Grade 1-2 |  |  | Grade 3-4 |  |  |
|  | No. of studies | Rate (95% CI) | I^2^ (%) | No. of studies | Rate (95% CI) | I^2^ (%) | No. of studies | Rate (95% CI) | I^2^ (%) |
| Total | 20 | 82.3% (73.9%-90.7%) | 94 | 3 | 36.5% (22.5%-50.5%) | 95 | 20 | 37.7% (26.9%-48.5%) | 97 |
| Dysphonia | 4 | 32.1% (11.3%-52.9%) | 91 | NA | NA | NA | NA | NA | NA |
| Hypertension | 13 | 31.1% (19.1%-43.2%) | 96 | 9 | 12.6% (7.7%-17.5%) | 93 | 10 | 14.7% (6.2%-23.1%) | 89 |
| Proteinuria | 11 | 30.4% (19.9%-40.9%) | 97 | 7 | 21.5% (12.0%-31.0%) | 96 | 11 | 3.4% (2.4%-4.5%) | 49 |
| Hand-foot syndrome | 6 | 28.0% (7.8%-48.2%) | 96 | NA | NA | NA | 5 | 0% (0%-0.4%) | 50 |
| ALT increased | 8 | 27.5% (13.4%-41.6%) | 96 | 4 | 19.8% (11.7%-27.9%) | 92 | 6 | 3.3% (0%-6.7%) | 81 |
| Platelet count decrease | 9 | 26.2% (10.9%-41.4%) | 96 | 7 | 14.6% (6.8%-22.4%) | 93 | 8 | 3.6% (0.9%-6.2%) | 80 |
| Fatigue | 10 | 25.5% (18.8%-32.3%) | 81 | 8 | 14.6% (7.2%-21.9%) | 95 | 7 | 1.7% (0.9%-2.6%) | 40 |
| Decreased Appetite | 13 | 24.9% (18.1%-31.7%) | 88 | 9 | 15.0% (7.5%-22.5%) | 95 | 9 | 0.9% (0.3%-1.4%) | 18 |
| AST increased | 14 | 24.6% (15.4%-33.8%) | 95 | 8 | 15.0% (7.3%-22.6%) | 95 | 11 | 4.9% (2.9%-7.0%) | 67 |
| Diarrhea | 9 | 24.4% (15.5%-33.2%) | 96 | 8 | 13.1% (7.9%-18.4%) | 84 | 9 | 1.8% (1.1%-2.5%) | 43 |
| Anemia | 3 | 20.3% (10.7%_29.9%) | 70 | NA | NA | NA | 3 | 2.2% (0.8%-3.7%) | 2 |
| Asthenia | 8 | 18.8% (10.8%-26.7%) | 93 | 3 | 10.6% (3.3%-18.0%) | 94 | 5 | 2.1% (0%-4.3%) | 84 |
| Gamma-glutamyltransferase increased | 4 | 18.5% (11.3%-25.7%) | 53 | NA | NA | NA | 4 | 8.9% (2.6%-15.2%) | 65 |
| Hypothyroidism | 10 | 16.8% (12.7%-20.9%) | 73 | 5 | 13.6% (5.2%-22.1%) | 96 | NA | NA | NA |
| Blood bilirubin increase | 6 | 16.7% (9.6%-23.9%) | 89 | NA | NA | NA | 5 | 2.3% (0.5%-4.0%) | 64 |
| Rash | 14 | 16.4% (12.3%-20.6%) | 76 | 8 | 11.6% (7.1%-16.0%) | 82 | 9 | 0.4% (0%-0.9%) | 0 |
| Weight decreased | 5 | 16.1% (12.2%-19.9%) | 65 | 3 | 13.3% (11.1%-15.5%) | 35 | 5 | 1.8% (0%-3.8%) | 84 |
| Pyrexia | 8 | 15.6% (13.1%-18.0%) | 31 | 6 | 11.7% (5.5%_17.8%) | 93 | 6 | 1.2% (0.1%-2.2%) | 55 |
| Abdominal pain | 8 | 14.6% (11.4%-17.8%) | 55 | 6 | 11.4% (7.5%-15.2%) | 66 | 7 | 0.8% (0%-1.6%) | 54 |
| Nausea | 6 | 12.2% (7.8%-16.7%) | 77 | 4 | 10.2% (5.5%-14.8%) | 85 | 6 | 0.5% (0.1%-1.0%) | 0 |
| Constipation | 5 | 12.1% (7.8%-16.4%) | 68 | 4 | 11.4% (5.9%-16.9%) | 73 | 6 | 1.0% (0.4%-1.6%) | 0 |
| Pruritus | 9 | 11.8% (8.9%-14.6%) | 47 | 7 | 10.2% (6.0%-14.4%) | 81 | 8 | 0.1% (0%-0.4%) | 0 |
| Cough | 5 | 11.3% (8.7%-13.9%) | 18 | 4 | 7.6% (2.0%-13.3%) | 75 | 4 | 0.6% (0%-1.5%) | 47 |
| Arthralgia | 4 | 11.1% (7.2%-15.0%) | 16 | NA | NA | NA | NA | NA | NA |
| Infusion-related reaction | 3 | 10.8% (7.7%-13.8%) | 0 | NA | NA | NA | NA | NA | NA |
| Peripheral edema | 4 | 10.1% (6.4%-13.8%) | 30 | 3 | 10.4% (4.6%-16.1%) | 53 | NA | NA | NA |
| Ascites | 3 | 9.0% (2.8%-15.3%) | 91 | NA | NA | NA | NA | NA | NA |
| Vomiting | 4 | 8.2% (5.3%-11.1%) | 56 | NA | NA | NA | NA | NA | NA |
| Alopecia | 4 | 5.2% (0%-13.6%) | 90 | NA | NA | NA | NA | NA | NA |
| Amylase increased | 3 | 2.6% (0%-6.9%) | 46 | NA | NA | NA | 3 | 0.4% (0%-3.4%) | 0 |

Abbreviations: ALT, alanine aminotransferase; AST, aspartate aminotransferase; NA, not available.

**A** Median PFS

**
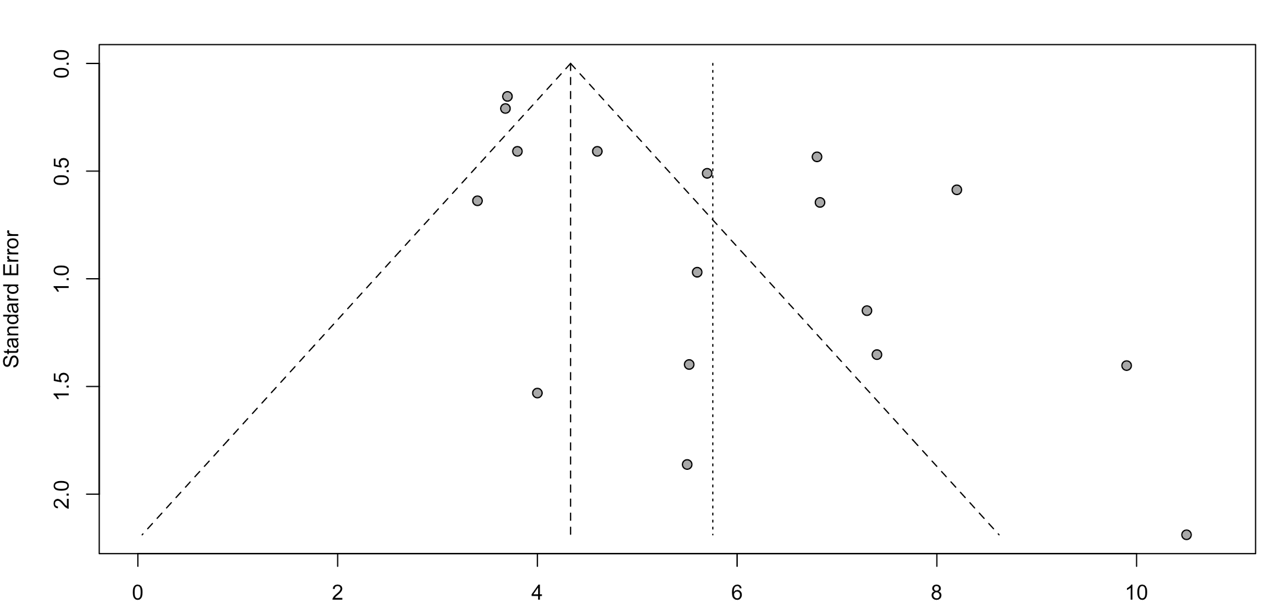
**

**B** Median OS

**
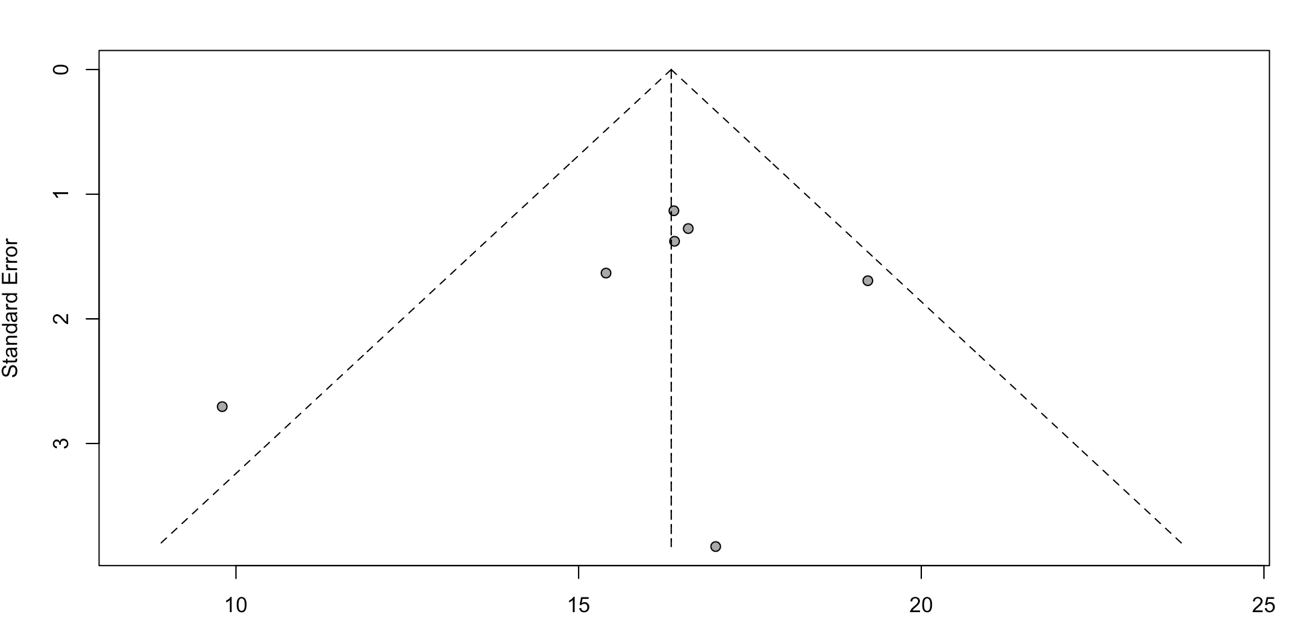
**

**Supplementary Fig 1. Funnel plots of PFS and OS**

**A** CR judged by RECIST v1.1**
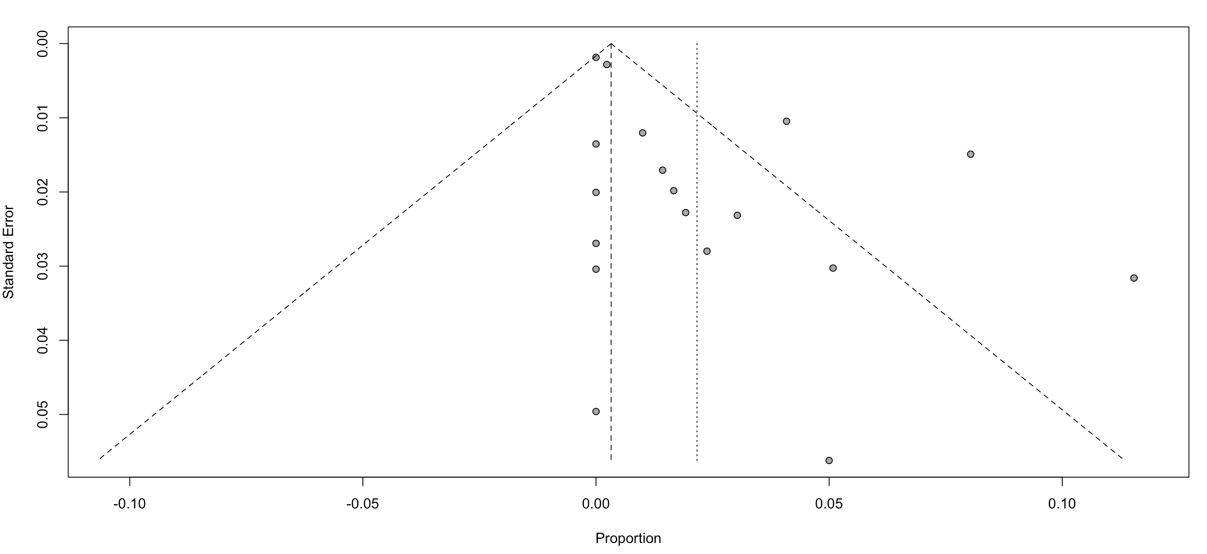
**

**B** PR judged by RECIST v1.1


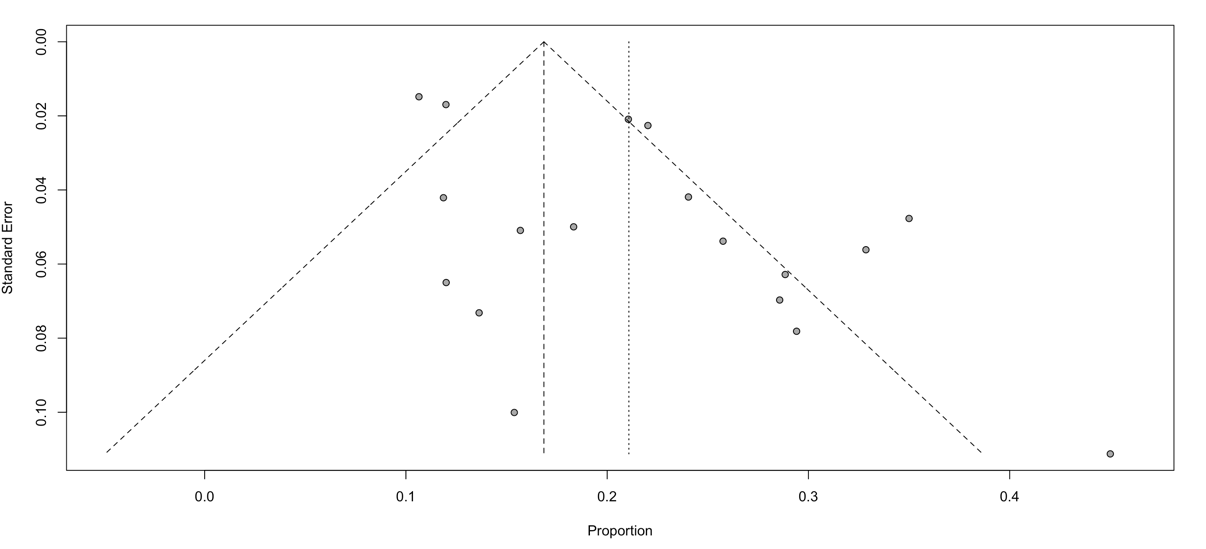


**C** SD judged by RECIST v1.1

**
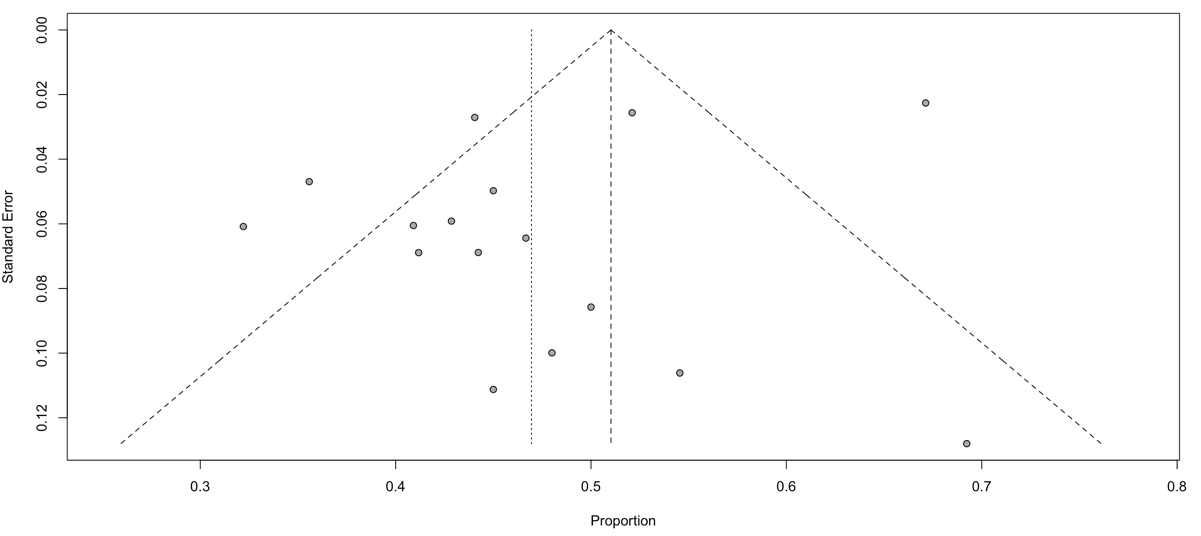
**

**D** PD judged by RECIST v1.1


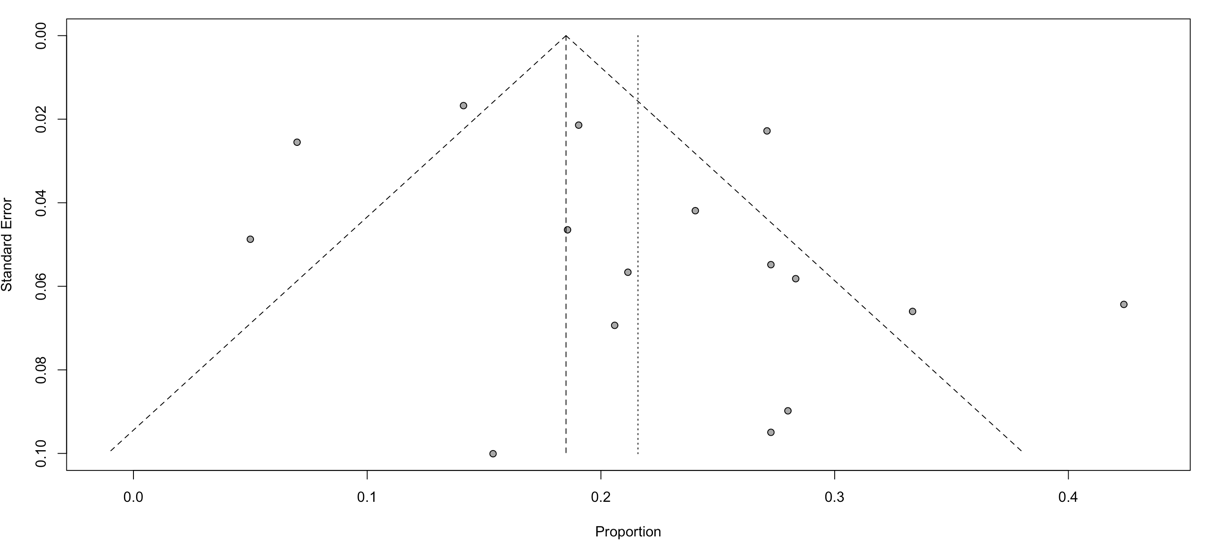


**E** ORR judged by RECIST v1.1


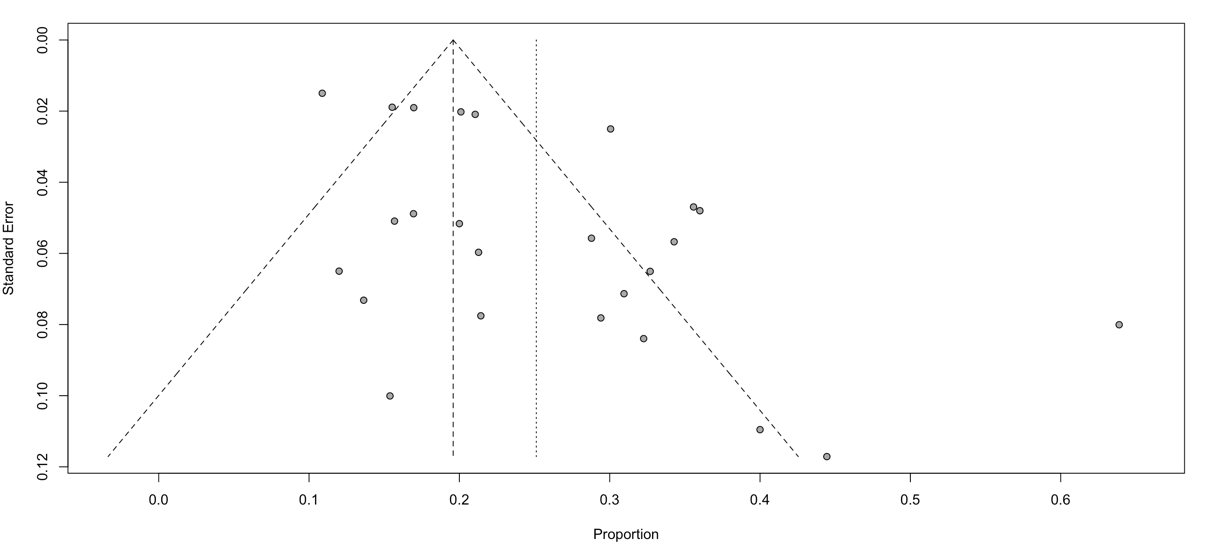


**F** DCR judged by RECIST v1.1


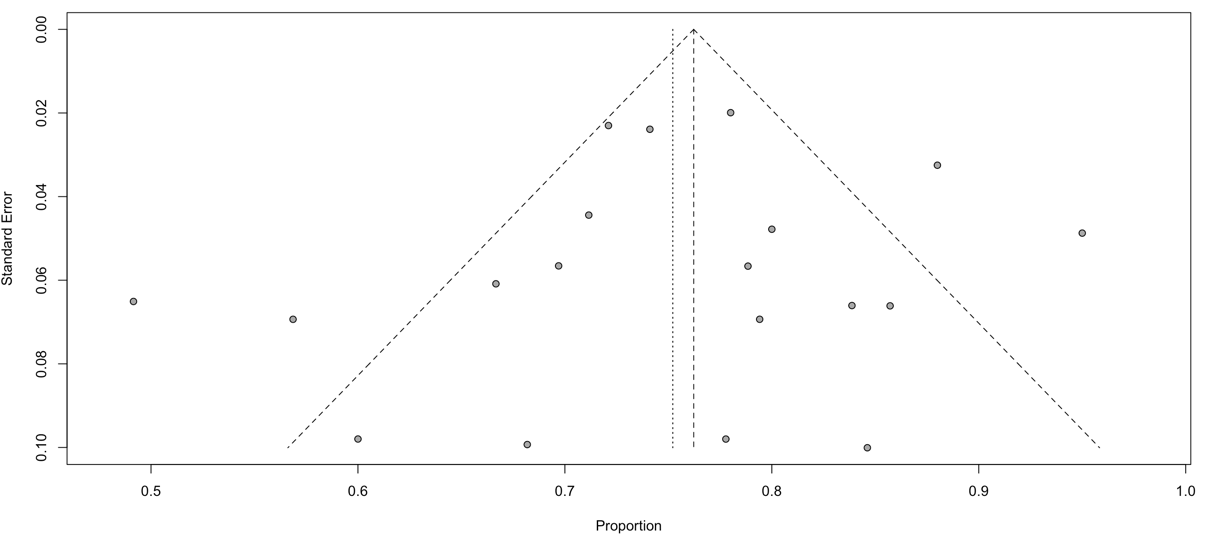


**G** CR judged by mRECIST v1.1


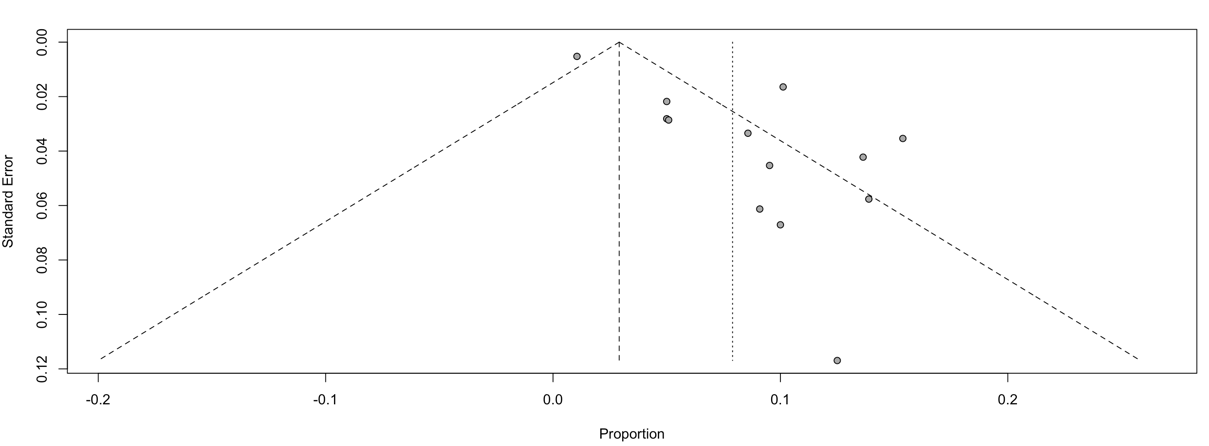


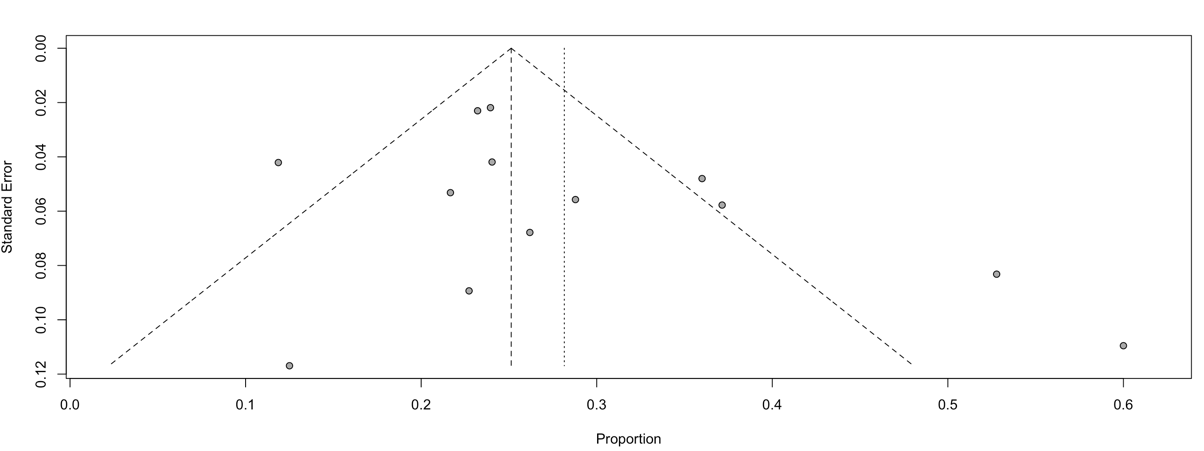
**H** PR judged by mRECIST v1.1

**I** SD judged by mRECIST v1.1


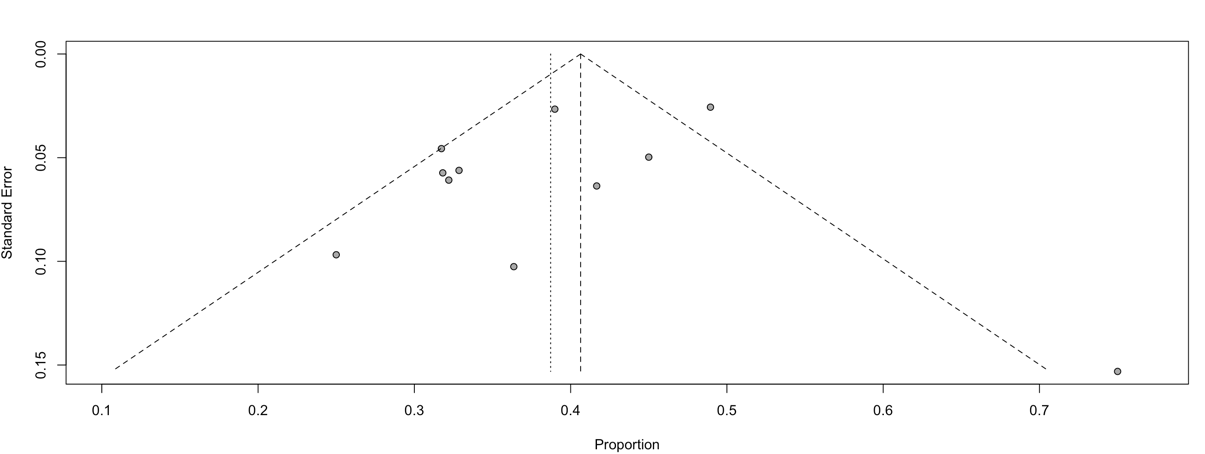


**J** PD judged by mRECIST v1.1
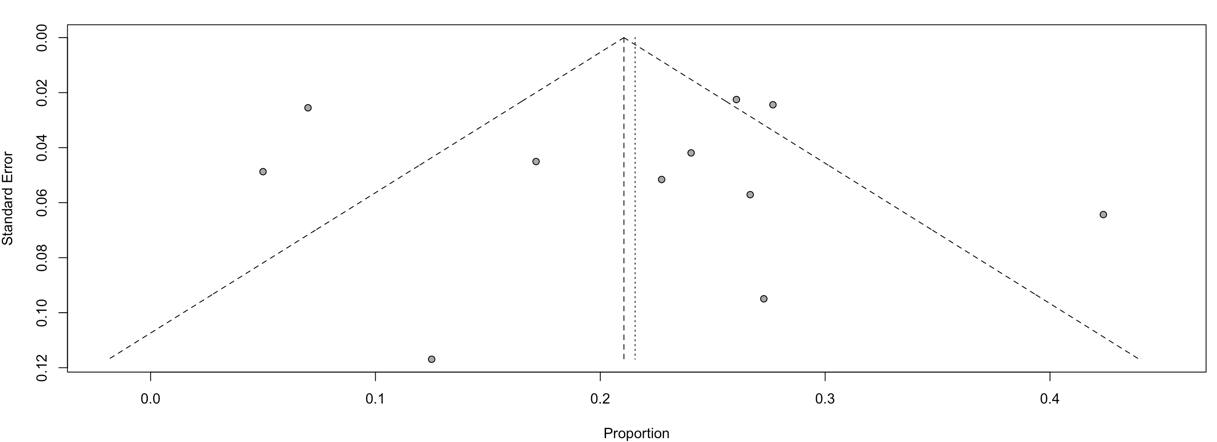


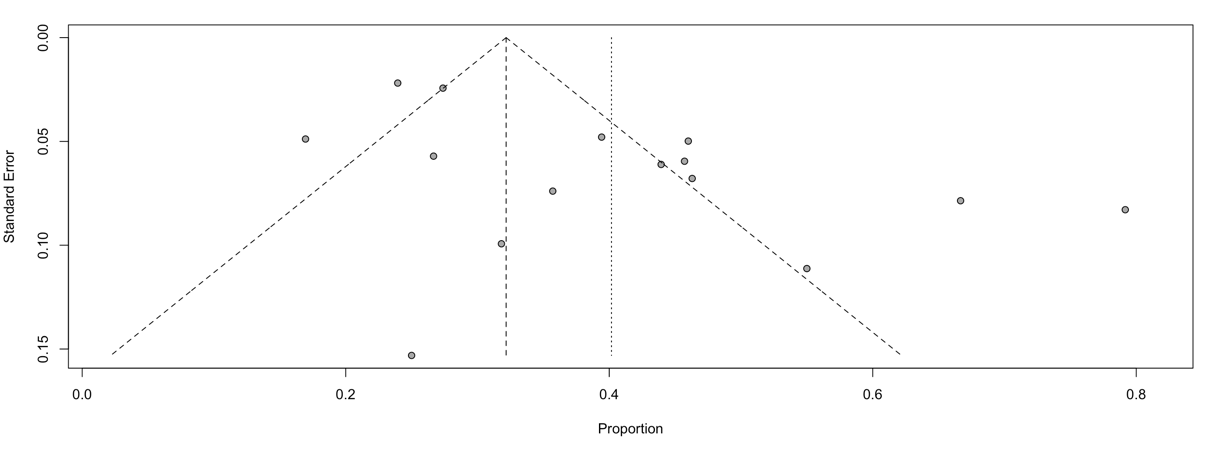
**K** ORR judged by mRECIST v1.1

**L** DCR judged by mRECIST v1.1


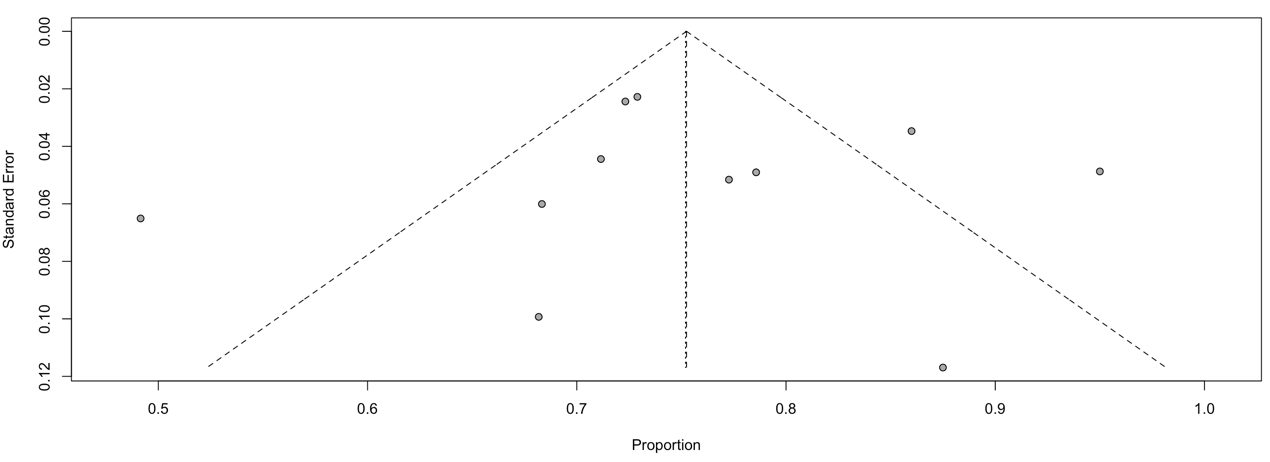


**Supplementary Fig 2. Funnel plots of best responses**

**Supplementary Table 3. Publication bias of PFS and OS (Egger’s test)**

| Outcome | Egger’s test |
| --- | --- |
| Median PFS | 0.0027 |
| Median OS | 0.4563 |

Abbreviations: OS, overall survival; PFS, progression-free survival.

**Supplementary Table 4. Publication bias of best responses (Egger’s test)**

| Outcome | RECIST v1.1 | mRECIST v1.1 |
| --- | --- | --- |
| CR | 0.0009 | 0.0082 |
| PR | 0.1481 | 0.0095 |
| SD | 0.3870 | 0.1029 |
| PD | 0.8974 | 0.1308 |
| ORR | 0.0099 | 0.0022 |
| DCR | 0.9418 | 0.6041 |

Abbreviations: CR, complete response; DCR, disease control rate; ORR, objective response rate; PD, disease progression; PR, partial response; SD, stable disease.
